# Intermolecular cascaded π-conjugation channels for electron delivery powering CO_2_ photoreduction

**DOI:** 10.1038/s41467-020-14851-7

**Published:** 2020-03-02

**Authors:** Shengyao Wang, Xiao Hai, Xing Ding, Shangbin Jin, Yonggang Xiang, Pei Wang, Bo Jiang, Fumihiko Ichihara, Mitsutake Oshikiri, Xianguang Meng, Yunxiang Li, Wakana Matsuda, Jun Ma, Shu Seki, Xuepeng Wang, Hao Huang, Yoshiki Wada, Hao Chen, Jinhua Ye

**Affiliations:** 1 College of Science, Huazhong Agricultural University, Wuhan, 430070 P. R. China; 2 International Center for Materials Nanoarchitectonics (WPI-MANA), National Institute for Materials Science (NIMS), 1-1 Namiki, Tsukuba, Ibaraki 305-0044 Japan; 3 Graduate School of Chemical Sciences and Engineering, Hokkaido University, Sapporo, 060-0814 Japan; 4 Key Laboratory of Material Chemistry for Energy Conversion and Storage, Ministry of Education, School of Chemistry and Chemical Engineering, Huazhong University of Science and Technology, Wuhan, 430074 P. R. China; 5 International Center for Material Nanoarchitectnoics (WPI-MANA), National Institute for Materials Science (NIMS), 3-13 Sakura, Tsukuba, Ibaraki, 305-0003 Japan; 6 Department of Molecular Engineering, Kyoto University, Kyoto, 615-8510 Japan; 7 Electroceramics Group, National Institute for Materials Science (NIMS), 1-1 Namiki, Tsukuba, Ibaraki 305-0044 Japan; 8 TJU-NIMS International Collaboration Laboratory, School of Materials Science and Engineering, Tianjin University, Tianjin, 300072 P. R. China

**Keywords:** Heterogeneous catalysis, Solar energy, Photocatalysis

## Abstract

Photoreduction of CO_2_ to fuels offers a promising strategy for managing the global carbon balance using renewable solar energy. But the decisive process of oriented photogenerated electron delivery presents a considerable challenge. Here, we report the construction of intermolecular cascaded π-conjugation channels for powering CO_2_ photoreduction by modifying both intramolecular and intermolecular conjugation of conjugated polymers (CPs). This coordination of dual conjugation is firstly proved by theoretical calculations and transient spectroscopies, showcasing alkynyl-removed CPs blocking the delocalization of electrons and in turn delivering the localized electrons through the intermolecular cascaded channels to active sites. Therefore, the optimized CPs (N-CP-D) exhibiting CO evolution activity of 2247 μmol g^−1^ h^−1^ and revealing a remarkable enhancement of 138-times compared to unmodified CPs (N-CP-A).

## Abstract (web summary)

While conversion of CO_2_ to fuels may offer a bio-inspired means to renewably utilize fossil fuel emission, most materials demonstrate poor activities for CO_2_ reduction. Here, authors construct conjugated polymers that modulate photo-induced electron transfer to CO_2_ reduction catalysts.

## Introduction

Global carbon dioxide (CO_2_) emissions from burning fossil fuels reached 33 gigatons in 2017, twice the natural rate at which CO_2_ is adsorbed back into land and ocean sinks. Harnessing solar radiation holds the answer to reducing our dependence on fossil fuels and reducing greenhouse gas emissions. The utilization of photoexcited high-energy photoelectrons with the help of semiconductors to drive the energy conversion of CO_2_ to value-added green fuels is believed to be an effective strategy for promoting sustainable development in society^1–5^. Beyond conventional semiconductors, conjugated polymers (CPs), as a new class of organic semiconductors, is expected to be the next generation of multifunctional photocatalyst because of their versatile photophysical properties, intriguing electronic properties, and especially the adjustable monomer structure, endowing it with manageable light absorption and controllable electronic localization ability. Therefore, numerous studies on developing various strategies for capable of photocatalytic energy conversion over CPs have been carried out^6–8^. Since Cooper’s group first found a series of pyrene-based CPs active in hydrogen (H_2_) evolution under visible light irradiation with platinum as cocatalyst^9^, researchers have tried to anticipate to the application of such materials in CO_2_ photoreduction^10^. For CPs in photocatalytic CO_2_ reduction, the light conversion efficiency is rate-determined by the photoexcited electrons delivery from CPs to the surface loaded cocatalyst (adsorb and active CO_2_ molecule). However, a limitation still exists in finding an appropriate way to promote the delivery of photoexcited electrons to cocatalyst due to a higher energy barrier of the out-of-plane Ohm or Schottky contact than the intramolecular cascade between cocatalyst and CPs^11,12^.

Photoinduced intermolecular charge transfer through molecules by non-covalent interactions is a well-known efficient process in photochemistry^13^. To achieve kinetically favorable electron transfer from CPs to cocatalyst and make a breakthrough in CO_2_ photoreduction, an intermolecular cascaded channel between the CPs and cocatalyst is desirable to be established for oriented delivery of photoexcited electrons to overcome a lower energy barrier and a less carrier^14–17^. Transition metal bipyridine compounds with a π-conjugated structure, such as Co (II) bipyridine complexes have been recognized as one of the most active centers for adsorbing and activating CO_2_ molecular and even achieving photocatalytic CO_2_ reduction in the presence of some photosensitizer^18–21^. However, the photocatalytic CO_2_ reduction process is severely hampered due to the instability of the light-absorbing material and the obstruction of interface electron transport^17^. When analyzing the spatial structure of π-conjugated Co (II) bipyridine complexes cocatalyst appeared in the large π-conjugated pyrene-based CPs, the most striking feature is that the strong π–π interactions will self-assemble them into an intermolecular π–π stacking structure^22–25^. Inspired by this, we speculated that an electronic transmission channel could be built via the enhanced π-electronic cloud interactions to ensure the photoexcited electrons freely deliver from CPs to cocatalysts^26^. Furthermore, for CPs, the absence of unsaturated bond between two adjacent aromatic rings can not only reduce the steric repulsion (reducing the twist angle of adjacent aromatic rings) but also weaken the intramolecular conjugate interaction^27,28^. The weakened conjugate interaction between adjacent aromatic rings of CPs is expected to result in free-π-electrons localized that could improve its intermolecular cascading ability with Co (II) bipyridine complexes^29^.

To validate the above strategy, four goal-oriented materials including linear and net-like (Net-like materials are usually referred to as conjugated microporous polymers) CPs with simple structure^24,25^, but different π-conjugation are built by using Suzuki-Miyaura coupling instead of Sonogashira-Hagihara coupling in synthesis. From first-principles theoretical calculation and experimental data, we prove that the CPs without alkynyl groups strictly block the delocalization of photoexcited electrons due to the lack of intramolecular charge-transfer bridges, which in turn deliver the photoexcited electrons faster to Co (II) bipyridine complexes through the intermolecular cascaded channels, leading to a state-of-the-art CO_2_ photoreduction activity. This strategy constructs an efficient system of CO_2_ photoreduction over Co (II) bipyridine complexes and pyrene-based CPs with modification of both intramolecular and intermolecular conjugations. Our results also provide evidence and mechanism of enhanced charge transfer via the pathway of non-covalent interactions. The built-in intermolecular cascaded channels worked out the most critical challenge in the electron delivery from CPs to cocatalyst, providing a point of view in the construction of CPs-based system for CO_2_ photoreduction.

## Results

### Synthesis, structure, and spectroscopic properties of CPs

**Fig. 1:**
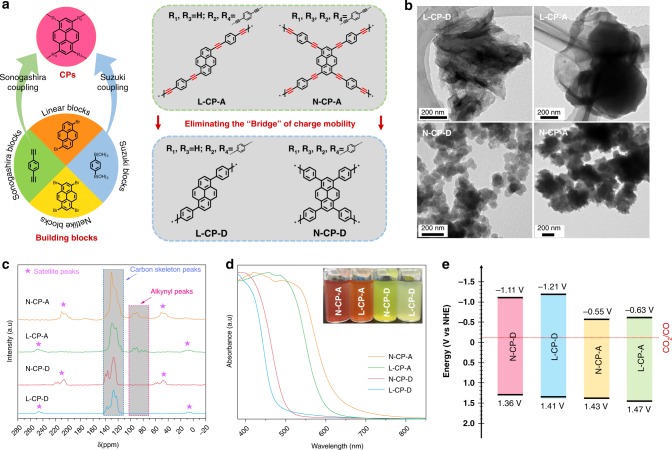
Preparation and characterization of the CPs. **a** Illustration of synthesis and the strategy of eliminating the charge-transfer bridge. **b** TEM images of CPs. **c** Solid-state ^13^C corss-polarization/magic angel spinning nuclear magnetic resonance (CP/MAS NMR) spectroscopy of CPs. **d** Solid-state UV/Visible diffuse reflectance spectra (DRS) of CPs, Inset: CPs dispersed in an acetonitrile/water (7:3) mixture. **e** Highest occupied molecular orbital (HOMO) and lowest unoccupied molecular orbital (LUMO) band position diagram for CPs obtained from cyclic voltammetry (CV) and DRS.

The four kinds of pyrene-based CPs with or without alkynyl groups were designed based on classic Sonogashira-Hagihara and Suzuki-Miyaura coupling processes, and linear or net-like structures were modified by changing the building blocks (see the “Methods” section for experimental details), as outlined in Fig. 1a^9,30^. Linear (L-CP-A) and net-like (N-CP-A) alkynyl-connected CPs can be obtained by using the Sonogashira blocks (1,4-diethynylbenzene) polymerized with the linear blocks (1,6-dibromopyrene) and net-like blocks (1,3,6,8-tetrabromopyrene), respectively (Supplementary Fig. 1). As a comparison, Suzuki blocks (1,4-phenylenediboronic acid) were employed as substitutes for Sonogashira blocks and successfully construct the corresponding linear (L-CP-D) and net-like (N-CP-D) directly connected CPs without alkynyl groups.

The transmission electron microscopy (TEM) and scanning electron microscopy (SEM) revealed that the CP-A series (L-CP-A and N-CP-A) exhibited a more agglomerated state than did the CP-D series (D-CP-A and D-CP-A) because of the higher π conjugation. (Fig. 1b and Supplementary Fig. 2). Although the CPs presented different agglomeration states at low resolution, the high-resolution transmission electron microscopy (HR-TEM) and the powder X-ray diffraction profile (PXRD) revealed that each CPs exhibits the basic characteristics of amorphous carbon, which means similar structure of CPs were constructed during the synthesis process (Supplementary Figs. 3 and 4)^31^. For a more in-depth comparison of the structural differences in CPs, we utilized solid-state ^13^C cross-polarization/magic angle spinning nuclear magnetic resonance (^13^C CP/MAS NMR) spectroscopy to demonstrate the exact structure (Fig. 1c)^32^. Referencing the estimated chemical shifts of different carbons in CPs (Supplementary Fig. 5), the similar chemical shifts of all these CPs between 110 and 130 parts per million (ppm) can be assigned to the aromatic carbons of the phenyl and pyrenyl units^33^. The peaks at ~140 ppm, which only existed in the CP-D series, were ascribed to the mutually substituted aromatic carbon. For the CP-A series, there are some signals at ~90 ppm can be indexed to the characteristic peak of alkynyl^34^. Notably, the locations of the satellite peaks attribute to spinning sidebands in N-CP-A were consistent with those in N-CP-D. A similar phenomenon was also found for L-CP-A and L-CP-D, which may be attributed to the structural difference between linear and net-liked CPs. Besides, the CPs with or without alkynyl was also confirmed by Raman spectroscopy, X-ray photoelectron spectroscopy (XPS) and Fourier transform infrared (FT-IR) spectroscopy (Supplementary Figs. 6–8)^35,36^.

After fully identifying the structure of CPs, we employed UV–Visible diffuse reflectance spectra (DRS) to monitor the light absorption of CPs^37^. As shown in Fig. 1d, the absorption band edges of CPs were located in the visible region ranging from 470 to 620 nm, which was consistent with the color of the CPs (inset of Fig. 1d). According to the Kubelka-Munk equation, the absorption band edges of L-CP-A and N-CP-A correspond to bandgaps of 2.10 and 1.98 eV, respectively. While the bandgaps of L-CP-D and N-CP-D were estimated to be 2.62 and 2.47 eV, respectively, which is larger than CP-A series due to the decreased conjugation (Supplementary Fig. 9)^38^. Cyclic voltammetry (CV) measurements were also conducted, the highest occupied molecular orbital (HOMO) position can be determined by the irreversibility of the oxidation peaks because the irreversible oxidation process of the CPs at the impressed voltage (Supplementary Fig. 10) revealed different energy levels within the CPs (Supplementary Table 1)^39^. In addition, their energy levels were further investigated by the ultraviolet photoelectron spectroscopy (UPS) (Supplementary Fig. 11) and Mott-Schottky test (Supplementary Fig. 12), which showed a high accordance with the energy levels determined by CV measurement (Supplementary Table 2). Although the CP-D series exhibited higher lowest unoccupied molecular orbital (LUMO) levels than the CP-A series, all of these CPs had enough negative potentials to carry out the reduction of CO_2_ to CO (Fig. 1e).

### Charge mobility and intermolecular cascaded channel of CPs

**Fig. 2:**
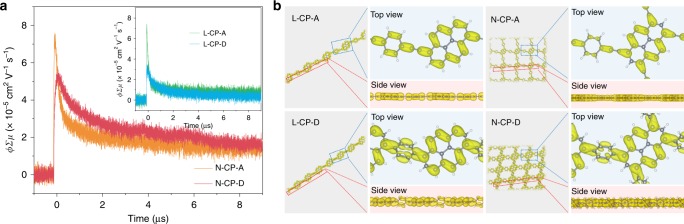
Characterization of electronic delocalization in CPs. **a** Conductivity transients observed by flash-photolysis time-resolved microwave conductivity (FP-TRMC) spectroscopy upon excitation at 355 nm laser pulses at 1.8 × 10^16^ photons cm^−2^ for CPs. **b** Top and side views of the charge distribution of LUMO of CPs at the Γ k-point.

Based on the conjugation of CPs, we can speculate the electron localization of CPs could be increased by eliminating the alkynyl group. For further confirmation of the weakened conjugate interaction and free-π-electrons localization of these CPs without the alkynyl group, the electrodeless flash-photolysis time-resolved microwave conductivity (FP-TRMC) was employed to evaluate photoexcited electrons transport in CPs. Unlike the conventional techniques that are highly affected by the influence of factors such as impurities, chemical or physical defects, and organic/electrode interfaces, FP-TRMC allows for probing the motion of the charge carrier before complete deactivation by trapping sites^40,41^. The conductivity transients and calculated charge mobilities for CPs are displayed in Fig. 2a, in which the L-CP-A, with a linear structure and alkynyl group, exhibits charge mobility (Σμ) of 0.32 cm^2^ V^−1^ s^−1^ (*ϕ*Σ*μ* = 7.4 × 10^−5^ cm^2^ V^−1^ s^−1^). As expected, the charge mobility of L-CP-D in the absence of alkynyl decreased to a much lower value of 0.15 cm^2^ V^−1^ s^−1^ (*ϕ*Σ*μ* = 3.4 × 10^−5^ cm^2^ V^−1^ s^−1^). For N-CP-A CPs, the network structure provided two additional pathways than that of linear structured L-CP-A for electronic delocalization due to the two more connections of the alkynyl group to each pyrenyl units, thus resulting in the maximum value of 0.35 cm^2^ V^−1^ s^−1^ (*ϕ*Σ*μ* = 7.6 × 10^−5^ cm^2^ V^−1^ s^−1^) among all CPs. In comparison, the N-CP-D possesses relative lower charge mobility of 0.25 cm^2^ V^−1^ s^−1^ (*ϕ*Σ*μ* = 5.5 × 10^−5^ cm^2^ V^−1^ s^−1^) as a result of the weak intramolecular conjugate interaction. Moreover, the CPs were subjected to additional electrochemical analyses, such as photocurrent measurement and the electrochemical impedance spectroscopy (EIS) Nyquist plots, which further validated that, consistent with the FP-TRMC results, the elimination of the alkynyl group in CPs greatly enhanced localization of free-π-electrons (Supplementary Figs. 13 and 14).

To gain insight into the effect of the alkynyl group on the localization of free-π-electrons, first-principles theoretical calculations based on hybrid functional were subsequently performed to compare the electronic localization of the CPs (see the “Methods” section for experimental details)^35^. As shown, the lowest unoccupied molecular orbital (LUMO) and highest occupied molecular orbital (HOMO) changes (Fig. 2b and Supplementary Fig. 15) at the Γ k-point distribution unambiguously demonstrated the apparent difference in the localization of free-π-electrons in CP-D series and CP-A series, respectively. From the top view of the charge distribution profiles of the two linear CPs (L-CP-A and L-CP-D), both LUMO and HOMO of L-CP-D exhibited stronger electron distribution asymmetry than those of L-CP-A as the conjugation changed. This means that L-CP-D could concentrate more electrons in a particular section under the light irradiation. Moreover, a similar phenomenon can also be observed from the comparison of two net-like CPs (N-CP-A and N-CP-D). In the light of the above results and electronic property comparison of backbone architecture with or without alkynyl (Supplementary Fig. 16), the energy gap between unoccupied and occupied orbital at the edge and corner of the pyrenyl is smaller than that at central area, the pyrenyl could play a role of a kind of antenna to collect the excited carriers at around the edge and corner. This is an advantage to achieve charge separation and harvest a wide range of photon energy.

Though the comparative analysis of computed interlayer interaction energies, it was consistency with what we expected (Supplementary Fig. 17 and Table 3). The existence of alkyne in CP-A series favors the electrons transfer in intramolecular. However, the CP-D series without alkynyl as a connector could possess more local photoelectrons, thus the interlayer interaction energy of bilayer CP-D series was much strong than that of CP-A series. This might indicate electron transfer along intermolecular of bilayer CP-D series more easily than that of bilayer CP-A series. Thus, we could propose the following situation on different CPs. For the CP-A series, photoelectrons were generated on the central light-responsive part of pyrenyl and then transferred to the other parts with alkynyl as a bridge, which results homogeneously charge density of CP-A series. In contrast, the absence of alkynyl in CP-D series led to a retardation of intramolecular electron delocalization. Moreover, it also gave rise to the increased charge density in some parts of CP-D series under light irradiation, which is favorable for improving the electronic delivery over the built-in intermolecular cascaded channels via π–π interactions between CPs and cocatalyst.

### CO_2_ Photoreduction activity over CPs

**Fig. 3:**
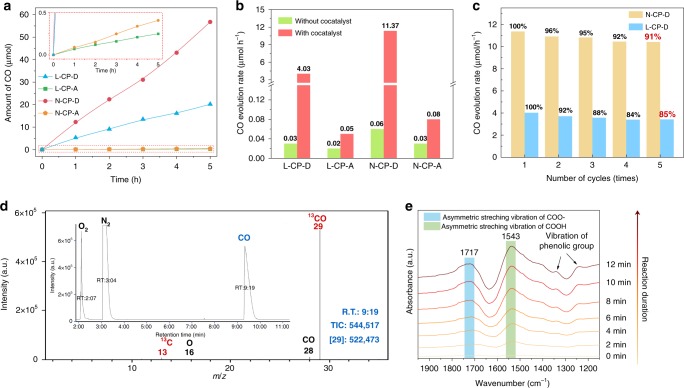
CO_2_ photoreduction performance of the CPs. **a** Time course of the produced CO for CPs during 5 h experiment performed under visible light (420 nm cut-off filter) in an acetonitrile/water (7:3) mixture using triethanolamine (TEOA) as sacrificial agent and 5 μmol Co (II) bipyridine complexes as a cocatalyst over 5 mg CPs under ~80 KPa of pure CO_2_ gas, inset: *X*-axis enlarged performance of CO evolution. **b** Average CO evolution rates for CPs with (red bar) or without (green bar) cocatalyst, **c** Recyclability of N-CPD (yellow bar) and L-CP-D (blue bar) in the photocatalytic CO_2_ reduction within five cycles. **d** Mass spectra of ^13^CO and total ion chromatography (inset) over N-CP-D in the photocatalytic reduction of ^13^CO_2_. **e** In-situ diffuse reflectance infrared Fourier transform spectroscopy (DRIFTS) for the photocatalytic reduction of CO_2_ over N-CP-D.

To study if the electron delivery from the CPs to cocatalyst has critical effects on CO_2_ photoreduction properties, the evaluation of CO_2_ photoreduction activities (see the “Methods” section for experimental details) were carried out in a closed gas circulation system by using CPs as the catalyst and 5 μmol Co (II) bipyridine complexes as cocatalyst. The acetonitrile/water (7:3) mixture with triethanolamine (TEOA) as sacrificial agent were also added. (Supplementary Fig. 18)^42,43^. The Co (I) bipyridine complexes produced by reduction of Co (II) bipyridine complexes are very powerful reducing agents which could be excellent candidates cocatalyst for photoreduction reaction based on the previous report^43^. We also demonstrated that photo-excited electrons on the LUMO of CPs in the present work do have the ability to reduce Co (II) bipyridine complexes to Co (I) bipyridine complexes via the cyclic-voltammetry spectrum (Supplementary Fig. 19). In addition, the weakened EPR signal (Supplementary Fig. 20, see the “Methods” section for experimental details)^22^ indicates the weakening of high-spin-state Co (II) upon visible-light irradiation that maybe reduced to low-spin-state Co (I). In order to get more convincing evidence for the existence of the low-spin-state of Co (I), the in-situ XPS (Supplementary Fig. 21) were performed over N-CP-D. It showed a lower binding energy than that of Co^2+^, which clearly revealed the existence of photo-induced low-spin-state Co^+^ in this system. In some recent researches, the Co (II) bipyridine complexes were selected as the model cocatalyst to study the CO_2_ photoreduction activity of the materials^17,42^. Therefore, we speculate that after optimizing the π-conjugation of CPs, the system consist of CPs and Co (II) bipyridine complexes will exhibit good potential for CO_2_ photoreduction. As displayed in Fig. 3a, after 5 h of visible light (>420 nm) irradiation, the N-CP-D generated a maximum amount of carbon monoxide (CO) of 56.8 μmol (11.37 μmol h^−1^), while the L-CP-D generated 20.2 μmol (4.03 μmol h^−1^) of CO. In contrast, only 0.3 and 0.4 μmol of CO was detected when L-CP-A and N-CP-A were used as the catalyst, respectively. The CO evolution rates of N-CP-D and L-CP-D were almost 138 times and 81 times higher than those of N-CP-A and L-CP-A, respectively. These CO_2_ photoreduction rates and enhancements are comparable to that of many similar systems containing photocatalyst and cobalt-complexes cocatalyst, especially π-conjugated Co (II) bipyridine complexes. It can be found that, although the addition of Co (II) bipyridine complexes significantly improved the photocatalytic CO_2_ reduction activity of the original photocatalyst, the photocatalytic CO_2_ reduction activity severely hampered by the obstruction of interface electron transport caused a relative lower multiple of increases (Supplementary Table 4). Owing to the solution of the oriented electron delivery, the activity of CO_2_ reduction reaction over the CPs with optimized conjugation showed a most considerable enhancement.

To further uncover the underlying reasons for the substantial difference that we obtained between the CP-D series and the CP-A series, we applied CPs in the CO_2_ photoreduction without a cocatalyst (Fig. 3b). All CPs showed quite low activity in the absence of cocatalyst, as well as the CP-D series in the presence of isolated cobalt chloride or dipyridyl (Supplementary Fig. 22), implying that the residual Pd has negligible effect on the photocatalytic CO_2_ reduction activity (Supplementary Table 5). After the addition of Co (II) bipyridine complexes as a cocatalyst, although all of CPs exhibit enough negative potential to transfer the photoelectrons to cocatalyst, only an ~2.5-fold increase was found for L-CP-A and N-CP-A. However, for L-CP-D and N-CP-D, the CO evolution rate increased by 134 times and 190 times, respectively. With the addition of appropriate cocatalyst (Supplementary Table 6), the CO selectivities of L-CP-D and N-CP-D were measured to be 86% and 82% (Supplementary Fig. 23) and achieved an apparent quantum yield (AQY) as high as 3.39% and 1.23% at 400 nm, respectively (Supplementary Fig. 24), which is considered higher than that in most reports until now (Supplementary Table 4). Moreover, the stability test over L-CP-D and N-CP-D indicates that after 5 cycles, the CO evolution was still high (maintaining 91% to the original cycle without adding fresh Co complexes to the system) as compared to the initial values (Fig. 3c), suggesting the adequate stability of these CPs (Supplementary Fig. 25). Therefore, we speculate that upon eliminating the alkynyl group in the structure of CPs, the weakened intramolecular conjugation of CP-D series blocks the delocalization of photoexcited electron. Stacked electrons on CP-D series were not well dispersed and thus tended to be fast delivered to cocatalyst through the intermolecular cascaded channels, making the CP-D series performed far better activity than did the CP-A series (Supplementary Fig. 26). Besides, the net-like CPs (N-CP-A and N-CP-D) possess more cocatalyst absorption sites of phenyl in the units, thus resulting in enhanced performance of net-like CPs (N-CP-A and N-CP-D) than those of linear CPs (L-CP-A and L-CP-D).

To validate the generation of CO originated from the catalytic splitting of CO_2_, we employed isotope-labeled carbon dioxide (^13^CO_2_) as a substitute source gas with the N-CP-D (Fig. 3d) or L-CP-D (Supplementary Fig. 27) to complete the evaluation experiment (see the “Methods” section for experimental details)^44^. As shown in Fig. 3d, the total ion chromatographic peak around 9.3 min can be assigned to the CO (inset of Fig. 3d). Moreover, the main peak at *m/z* = 29 achieved a high abundance, and the fragments produced from this peak (^13^C at *m/z* = 29 and O at *m/z* = 16) in the mass spectra indicated that the produced ^13^CO indeed originated from ^13^CO_2_ in the CO_2_ photoreduction over N-CP-D. A similar phenomenon can also be observed in using L-CP-D as catalyst and intensity of ^13^CO is one-third of using N-CP-D (Supplementary Fig. 27), which is highly consistent with the above activity measurement. Subsequently, we also used the in-situ diffuse reflectance infrared Fourier transform spectroscopy (DRIFTS) to unveil the process of CO_2_ photoreduction occurring on N-CP-D with a cocatalyst (see the “Methods” section for experimental details). Based on the spectra collected for the N-CP-D with or without absorbed CO, the in situ generated CO increased with the illumination time (Supplementary Fig. 28) and the main intermediates of this process were assigned to the asymmetric stretching vibrations of COO^−^ and COOH species in Fig. 3e^45^, which is solidly in accordance with the mechanism of CO_2_ conversion to CO in the previous report^46,47^.

### Electron delivery from CPs to cocatalyst for CO_2_ reduction

**Fig. 4:**
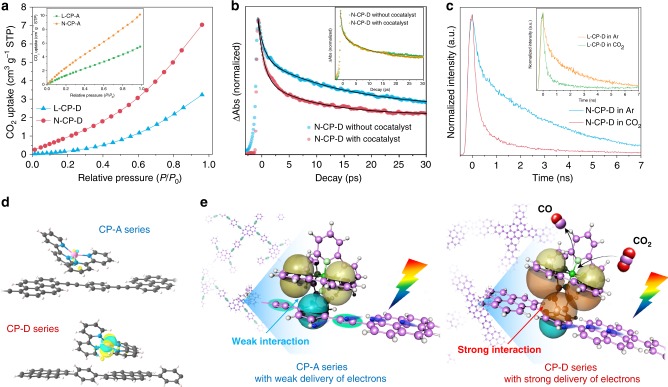
Electron delivery from CPs to cocatalyst for CO2 photoreduction. **a** CO_2_ adsorption capacities of the CP-D series and CP-A series (inset) at 273.15 K. **b** Kinetics of electrons in transient absorption over N-CP-D with or without cocatalyst under a probe wavelength of 2200 nm, inset: Kinetics of electrons in transient absorption over N-CP-A with or without cocatalyst under a probe wavelength of 2500 nm. **c** Time-resolved photoluminescence decay of L-CP-D and N-CP-D (inset) under a CO_2_ or argon atmosphere. **d** Charge density difference of CPs in present and absent of Co (II) bipyridine complexes with the isosurfaces value of 0.001 e/Å^3^. yellow and cyan represent charge accumulation and charge depletion, respectively. The pink, indigo, dark gray and white pink balls represent Co, N, C and H atoms, respectively. **e** Proposed process of electron transfer over the CP-A series and CP-D series for the CO_2_ photoreduction reaction.

By discovering and scrutinizing the CO_2_ photoreduction of CPs, we hope to verify our conjecture that the localized free-π-electrons of CPs could improve its intermolecular cascading ability with cocatalyst for CO_2_ photoreduction. The adsorption of CO_2_ is a prerequisite for CO_2_ photoreduction. Although the CP-D series have a larger specific surface area (Supplementary Fig. 29 and Table 7) due to a shorter skeleton length^48^, the CP-A series exhibited greater CO_2_ adsorption than the CPs-D series (Fig. 4a), attributing to the high conjugation from bifunctionalization with pyrenyl and alkynyl group^49^. Nevertheless, the CO_2_ absorption of a dry powder is very different from the wet conditions^50^, the in-situ FT-IR indicates L-CP-D and N-CP-D exhibit enhancement both in CO_2_ adsorption and CO_2_ chemisorption than L-CP-A and N-CP-A (Supplementary Fig. 30). It means that the CO_2_ adsorption capacity of CPs-D series is stronger than that of CPs-A series under the solvent-containing environment, which provides favorable conditions for the subsequent CO_2_ reduction reaction. Femtosecond transient absorption (TA) spectroscopy is a useful technique for studying ultrafast charge transfer in interfaces. We thus employed this method (see the “Methods” section for experimental details) to verify the different kinetics in intermolecular electron delivery for the solid evidence of built-in intermolecular cascade channel. From the TA spectra dependence on the wavelength (Supplementary Fig. 31), the main state of trapped electrons can be assigned to the wavelength of 2500 and 2200 nm, while the corresponding state of holes at 550 and 525 nm for N-CP-A and N-CP-D, respectively. Considering these results, we measured the kinetics of electrons over N-CP-D with or without cocatalyst under a probe wavelength of 2200 nm. In Fig. 4b, compared to the initial one, the decay of N-CP-D with cocatalyst showed a noticeable decrease, while N-CP-A showed no difference with or without cocatalyst (inset of Fig. 4b). Combining the results for the corresponding holes (Supplementary Figs. 32 and 33), the enhanced electron delivery over N-CP-D was determined. Moreover, we employed time-resolved photoluminescence (TR-PL) spectroscopy to gain insight into the subsequent process of electrons transfer from the cocatalyst to CO_2_ (see the “Methods” section for experimental details)^51^. For both N-CP-D (Fig. 4c) and L-CP-D (inset of Fig. 4c), the average lifetime of electrons (Supplementary Table 8) in a CO_2_ atmosphere was significantly shortened compared to that in an argon atmosphere, which can be mainly attributed to the large amount of photogenerated electrons can be fast delivered to CO_2_ via the cocatalyst and only a small number of electrons with short lifetime involved into the detectable recombination process under the CO_2_ atmosphere (Supplementary Fig. 34), which can be further confirmed by the TA spectra in different atmosphere (Supplementary Fig. 35). However, both N-CP-A and L-CP-A in TR-PL measurements showed no difference between the atmosphere of CO_2_ and argon due to the weak delivery (Supplementary Fig. 36).

In order to gain a deeper insight into the built-in intermolecular cascaded channels between CPs and Co (II) bipyridine complexes, the charge distribution analysis and charge density difference (Fig. 4d and Supplementary Figs. 37 and 38) of before and after adsorption of Co (II) bipyridine complexes over L-CP-D (0.12 e) and L-CP-A (0.07e) were used to figure out that more charges were delivered from the CP-D series to the adsorbed Co (II) bipyridine complexes than that of CP-A series. It indicates that the CP-A series in presence of alkynyl facilitate its intramolecular charge mobility, while the CP-D series in absence of alkynyl promote its intermolecular cascading ability with the Co (II) bipyridine complexes. Therefore, we can rationally propose that the CO_2_ photoreduction over CPs was promoted by the built-in intermolecular cascaded channels, as shown in Fig. 4e. When using the CP-A series, the photoelectrons were generated and quickly transferred to the other part of the CPs in the presence of alkynyl groups as the bridge. Since the electrons were distributed throughout the CPs, it was difficult to be delivered to the cocatalyst through the weak intermolecular π–π interactions in CP-A series. In contrast, by eliminating the alkynyl group, the CP-D series could prevent photoexcited electrons from transferring to other parts of CPs due to lack of intramolecular charge-transfer bridges. An intermolecular cascaded channel could be built via the enhanced π-electronic cloud interactions between CPs and cocatalyst to ensure the delivery of photoexcited electrons. As a result, a cascaded electron supply though the above intermolecular channel was fast delivered to the metal center of the cocatalyst, working out the most critical challenge in the oriented electron delivery from CPs to cocatalyst and achieves the efficient π-conjugation system for CO_2_ photoreduction (Supplementary Fig. 39)^12^.

## Discussion

To summarize, we found that an intermolecular cascaded channel for the electron delivery can be established through the modification of intramolecular and intermolecular π–π conjugation. Encouragingly, with the strategy of utilizing the Suzuki-Miyaura coupling instead of Sonogashira-Hagihara coupling to modify both intramolecular conjugation and intermolecular interaction of CPs, the photoelectrons generated from the pyrene part were localized on the benzene part of CPs, which in turn deliver the photoelectrons faster to Co (II) bipyridine complexes through the intermolecular cascaded channel. The current work reported directly connected net-like CPs systems exhibits highest CO evolution activity of 2247 μmol g^−1^ h^−1^ with an apparent quantum efficiency exceeds 3.39% among all reported CPs and most considerable enhancement of 138-times compared to unmodified CPs (N-CP-A) in CO_2_ photoreduction with adding Co (II) bipyridine complexes as cocatalyst. From the theoretical calculations and transient spectroscopy techniques, this high efficiency could be attribute to the intermolecular cascaded channels built by modification of π-conjugation in CPs. In addition, this strategy constructs a reliable system of CO_2_ photoreduction over CPs via smart engineering in molecular level, which figures out the most critical challenge in the oriented electron delivery as well as providing a viable route for designing high-efficiency polymers-based systems of CO_2_ photoreduction.

## Methods

### Synthesis of CP-A series (L-CP-A and N-CP-A)

All reagents were purchased from Sigma-Aldrich or Tokyo Chemical Industry without further purification. The CP-A series were synthesized according to Sonogashira-Hagihara cross-coupling polycondensation. In details, a dry 250 mL round-bottom flask was charged with two monomer reactants, Pd(PPh_3_)Cl_2_ and CuI, and mixed solvent of dimethyl formamide/triethylamine (DMF/TEA). The mixture was degassed by bubbling with Ar for 30 min, and then the resulting mixture was stirred at 80 °C for 24 h under Ar condition. After that, the precipitate was collected by filtration, and the solid was washed with methanol and CH_2_Cl_2_ in the Soxhlet for 48 h. The final product was dried at 60 °C overnight. For synthesis of L-CP-A, 1,4-diethynylbenzene (189 mg, 1.5 mmol), 1,6-dibromopyrene (540 mg, 1.5 mmol) Pd(PPh_3_)Cl_2_ (27 mg), CuI (5 mg), DMF (60 mL), and TEA (60 mL) were used. For N-CP-A, 1,4-diethynylbenzene (189 mg, 1.5 mmol), 1,3,6,8-Tetrabromopyrene (388 mg, 0.75 mmol), Pd(PPh_3_)Cl_2_ (27 mg), CuI (5 mg), DMF (60 mL), and TEA (60 mL) were used.

### Synthesis of CP-D series (L-CP-D and N-CP-D)

All reagents were purchased from Sigma-Aldrich or Tokyo Chemical Industry without further purification. The CP-D series were synthesized according to Suzuki-Miyaura cross-coupling polycondensation. In details, a dry 250 mL round-bottom flask was charged with two monomer reactants, Pd(PPh_3_)_4_, K_2_CO_3_, and mixed solvent of dimethyl formamide/water (DMF/H_2_O). The mixture was degassed by bubbling with Ar for 30 min and then the resulting mixture was stirred at 150 °C for 24 h under Ar condition. After that, the precipitate was collected by filtration, and the solid was washed with methanol and CH_2_Cl_2_ in the Soxhlet for 48 h. The final product was dried at 60 °C overnight. For synthesis of L-CP-D, 1,4-phenylenediboronic acid (248 mg, 1.5 mmol), 1,6-dibromopyrene (540 mg, 1.5 mmol), Pd(PPh_3_)_4_ (10 mg), K_2_CO_3_ (2.0 g) DMF (60 mL), and H_2_O (8 mL) were used. For N-CP-D, 1,4-phenylenediboronic acid (248 mg, 1.5 mmol), 1,3,6,8-Tetrabromopyrene (388 mg, 0.75 mmol), Pd(PPh_3_)_4_ (10 mg), K_2_CO_3_ (2.0 g) DMF (60 mL), and H_2_O (8 mL) were used.

### Characterization

Transmission electron microscopy (TEM, Tecnai G2 F20, FEI, Holland) and scanning electron microscope (SEM, SU8010, Hitachi, Japan) were used to analyze the morphologies of polymers. The diffractometer (D8 advance, Bruker, Germany) with Cu Kα radiation was used to record the Powder X-ray diffraction (PXRD). The solid-state ^13^C cross polarization magic angle spinning (^13^C-CP/MAS) NMR spectra (Avance III HD 400 MHz spectrometer, Bruker, Germany) were measured to analyze the structure of polymers. The infrared spectra were recorded using a Fourier transform-infrared (FT-IR) spectrometer (Nicolet 6700, Thermo Scientific, USA). Raman spectra were collected using a Raman spectrometer (DXR, Thermo Scientific, USA). The low-pressure gas adsorption measurements were investigated with a gas adsorption analyzer (ASAP 2040, Micrometrics, USA) using nitrogen/carbon dioxide as the adsorbate at 77 K. UV–Vis diffused reflectance spectra (DRS) were measured on a spectrometer (UV-3100, Shimadzu, Japan). The thermal stability was evaluated by thermal gravity analysis (TGA, TG 209 F3 Tarsus, Netzsch, Germany) with the temperature increased to 10 °C min^−1^ under air atmosphere. Metal content was determined by inductively coupled plasma mass spectrometry (ICP-MS, 7800, Agilent Technologies, USA), where the sample was first digested by H_2_SO_4_/HNO_3_ (0.8 mL/0.2 mL) solvent at 60 °C. The surface electronic states of polymers were analyzed via X-ray photoelectron spectroscopy (XPS, ESCALAB 250Xi, Thermo Scientific, USA). Cyclic voltammetry (CV) measurements, electrochemistry impedance spectroscopy (EIS) and photocurrent intensity response measurements were performed in a typical three-electrode cell system. Electrochemical measurements were carried out in a conventional three-electrode system using an Ag/Ag^+^ as reference electrode, platinum plate as the counter electrode, and the sample modified glassy carbon as the working electrode. The electrochemical workstation was a CHI 660E potentiostat (Shanghai Chenhua Co.) and CVs were collected at a scan rate of 50 mV s^−1^ with the protection of nitrogen. A solution of 0.1 M TBAPF_6_ in CH_3_CN was used as the electrolyte.

### FP-TRMC measurement

FP-TRMC measurements were carried out at room temperature under a N_2_ atmosphere, using different CPs powder on poly(methylmethacrylate) (PMMA) films. The films were cast onto quartz substrates. The microwave power and frequency were set at 3 mW and ~9.1 GHz, respectively. Charge carriers were generated in the films by direct excitation of CPs using third-harmonic generation (*λ* = 355 nm) light pulses from a Nd: YAG laser (Spectra Physics, INDI-HG). The excitation density was tuned at 1.8 × 10^16^ photons cm^−2^. The TRMC signal from a diode was recorded on a digital oscilloscope (Tektronix, TDS 3032B). Comparison of the integrated photocurrents with the polymer standard (poly-9,9′-dioctylfluorene, *ϕ* ~ 2.3 × 10^−4^) allowed determination of the quantum efficiency of charge carrier generation for the CPs samples. The local-scale charge carrier mobility Σμ was estimated by the quotient of (*ϕ*Σ*μ*) _max_ by *ϕ*.

### Photocatalytic activities measurement

The photoreduction CO_2_ activities of all CPs were carried out in gas-closed system with a gas-circulated pump. The setup of the photocatalytic system is illustrated in Fig. S17. In detail, the 5 mg catalyst, 50 mL of solution (acetonitrile/water = 7:3), 5 mL of TEOA and the synthesized Co (II) bipyridine complexes cocatalyst^43^ were added in a Pyrex glass reaction cell which was connected to the CO_2_ reduction system. After complete evacuation of the reaction system (no O_2_ or N_2_ could be detected by gas chromatography), ~80 kPa of pure CO_2_ gas was injected into the airtight system. After adsorption equilibrium, a 300 W xenon lamp (~100 mW/cm^2^) with a UV-cut filter (L42), to remove light with wavelengths lower than 420 nm (*λ* > 420 nm) was used as the light source. The produced H_2_ and CO was analyzed by two gas chromatographs (GC-8A and GC-2014, Shimadzu Corp., Japan) equipped with different chromatographic column^12,22^.

### Isotope labeling measurement

The isotope labeling measurement was carried out by using ^13^CO_2_ gas (Isotope purity, 99% and chemical purity, 99.9%, Tokyo Gas Chemicals Co., Ltd.) instead of pure ^12^CO_2_ gas (Chemical purity, 99.999%, Showa Denko Gas Products Co., Ltd.) as the carbon source with the same reaction set as mentioned above and the gas products were analyzed by gas chromatography-mass spectrometry (JMS-K9, JEOL-GCQMS, Japan and 6890N Network GC system, Agilent Technologies, USA) equipped with two different kinds of column for detecting the products of ^13^CO (HP-MOLESIEVE, 30 m × 0.32 mm × 25 μm, Agilent Technologies, USA) and source of ^13^CO_2_ (HP-PLOT/Q, 30 m × 0.32 mm × 20 μm, Agilent Technologies, USA), respectively^12,22^.

### In-situ DRIFTS measurement

In-situ DRIFTS measurement were carried out by FT-IR spectrometer (Nicolet iS50 Thermo Scientific, USA) with a designed reaction cell simulated in Fig. S23a. The substrate lying in the center of the designed reaction cell and a thin layer of N-CP-D mixture with or without cocatalyst as the model sample was placed uniformly on the substrate. An ultra-high vacuum pump was used to pump out all the gases in the reaction cell and adsorbed on the photocatalyst surface. Then the large amount of carbon dioxide or carbon monoxide was pumped in to construct a CO_2_ or CO atmosphere for CO_2_ photoreduction or CO adsorption, respectively. At last, visible light was turned on and the IR signal was in-situ collected through MCT detector along with the reaction.

### TA measurement

The output pulses of a 1 kHz Ti: sapphire regenerative amplifier (Solstice, Spectra-Physics) were split into two beams. One of the beams was used as an excitation light source of an optical parametric amplifier (OPA) (TOPAS prime, NIR-UV-Vis. LIGHT CONVERSION Inc.). The 420 nm output of the OPA was as the excitation light source. The excitation light was chopped at 500 Hz by an optical chopper. The other beam was used as an excitation light source of another OPA. The output of the OPA was used as the wavelength-tunable probe and reference light source (500–2600 nm). Si photodetectors and InGaAs photodetectors were used to detect the probe and reference light depending on probe light wavelength (500–1000 nm and 1000–2600 nm respectively). (Supplementary Fig. 40).

### TR-PL measurement

Photoluminescence spectra decay curves were obtained by using a Hamamatsu instrument (Hamamatsu C5680, Japan) with a 1 kHz Ti: sapphire regenerative amplifier (Solstice, Spectra-Physics) was used as an excitation light source of an OPA (TOPAS prime, NIR-UV-Vis. LIGHT CONVERSION Inc.). The CPs were dispersed in the solvent of acetonitrile and water solution with scavenger of TEOA. The argon was firstly pumped into a sealed cell for 40 min by using a pipe inlet to exclude the dissolved oxygen and other atmosphere. After the above pre-treatment process, the TR-PL signals were recorded and these samples are named L-CP-A in Ar, L-CP-D in Ar, N-CP-A in Ar and N-CP-D in Ar, respectively. As a comparison, the CO_2_ was subsequently pumped into this sealed cell for 40 min by using a pipe inlet. The TR-PL signals were also recorded, and these samples are named L-CP-A in CO_2_, L-CP-D in CO_2_, N-CP-A in CO_2_ and N-CP-D in CO_2_, respectively.

## Supplementary information

Supplementary Information

Peer Review File

## Data Availability

The data that support the plots within this paper and other findings of this study are available from the corresponding author upon reasonable request.

## source data

Source Data

## References

[1] Diercks CS, Liu Y, Cordova KE, Yaghi OM (2018). The role of reticular chemistry in the design of CO_2_ reduction catalysts. Nat. Mater..

[2] Niu K (2017). A spongy nickel-organic CO_2_ reduction photocatalyst for nearly 100% selective CO production. Sci. Adv..

[3] Li J (2018). Efficient electrocatalytic CO_2_ reduction on a three-phase interface. Nat. Catal..

[4] Studt F (2014). Discovery of a Ni-Ga catalyst for carbon dioxide reduction to methanol. Nat. Chem..

[5] Schreier M (2017). Solar conversion of CO_2_ to CO using Earth-abundant electrocatalysts prepared by atomic layer modification of CuO. Nat. Energy.

[6] Kim J, Swager T (2001). Control of conformational and interpolymer effects in conjugated polymers. Nature.

[7] Yu J, Hu D, Barbara PF (2000). Unmasking electronic energy transfer of conjugated polymers by suppression of O_2_ quenching. Science.

[8] Wang X (2009). A metal-free polymeric photocatalyst for hydrogen production from water under visible light. Nat. Mater..

[9] Sprick RS (2015). Tunable organic photocatalysts for visible-light-driven hydrogen evolution. J. Am. Chem. Soc..

[10] Yang C (2016). Molecular engineering of conjugated polybenzothiadiazoles for enhanced hydrogen production by photosynthesis. Angew. Chem. Int. Ed..

[11] Lin S (2015). Covalent organic frameworks comprising cobalt porphyrins for catalytic CO_2_ reduction in water. Science.

[12] Zhang H (2016). Efficient visible‐light‐driven carbon dioxide reduction by a single‐atom implanted metal-organic framework. Angew. Chem. Int. Ed..

[13] Fu M-C, Shang R, Zhao B, Wang B, Fu Y (2019). Photocatalytic decarboxylative alkylations mediated by triphenylphosphine and sodium iodide. Science.

[14] Ran J, Jaroniec M, Qiao SZ (2018). Cocatalysts in semiconductor‐based photocatalytic CO_2_ reduction: achievements, challenges, and opportunities. Adv. Mater..

[15] Rao H, Schmidt LC, Bonin J, Robert M (2017). Visible-light-driven methane formation from CO_2_ with a molecular iron catalyst. Nature.

[16] Costentin C, Robert M, Savéant J-M (2015). Current issues in molecular catalysis illustrated by iron porphyrins as catalysts of the CO_2_-to-CO electrochemical conversion. Acc. Chem. Res..

[17] Li X, Yu J, Jaroniec M, Chen X (2019). Cocatalysts for selective photoreduction of CO_2_ into solar fuels. Chem. Rev..

[18] Huang C (2015). Carbon-doped BN nanosheets for metal-free photoredox catalysis. Nat. Commun..

[19] Kuehnel MF, Orchard KL, Dalle KE, Reisner E (2017). Selective photocatalytic CO_2_ reduction in water through anchoring of a molecular Ni catalyst on CdS nanocrystals. J. Am. Chem. Soc..

[20] Zheng Y, Lin L, Ye X, Guo F, Wang X (2014). Helical graphitic carbon nitrides with photocatalytic and optical activities. Angew. Chem. Int. Ed..

[21] Wang S, Guan BY, Lou XWD (2018). Construction of ZnIn_2_S_4_-In_2_O_3_ hierarchical tubular heterostructures for efficient CO_2_ photoreduction. J. Am. Chem. Soc..

[22] Zhao G (2018). Efficient photocatalytic CO_2_ reduction over Co(II) species modified CdS in aqueous solution. Appl. Catal. B-Environ..

[23] Kuriki R, Sekizawa K, Ishitani O, Maeda K (2015). Visible‐light‐driven CO_2_ reduction with carbon nitride: enhancing the activity of ruthenium catalysts. Angew. Chem. Int. Ed..

[24] Cooper AI (2009). Conjugated microporous polymers. Adv. Mater..

[25] Xu Y, Jin S, Xu H, Nagai A, Jiang D (2013). Conjugated microporous polymers: design, synthesis and application. Chem. Soc. Rev..

[26] Maurin A, Robert M (2016). Noncovalent immobilization of a molecular iron-based electrocatalyst on carbon electrodes for selective, efficient CO_2_-to-CO conversion in water. J. Am. Chem. Soc..

[27] Noriega R (2013). A general relationship between disorder, aggregation and charge transport in conjugated polymers. Nat. Mater..

[28] Liu W (2017). A two-dimensional conjugated aromatic polymer via C-C coupling reaction. Nat. Chem..

[29] Leung JJ (2019). Solar-driven reduction of aqueous CO_2_ with a cobalt bis(terpyridine)-based photocathode. Nat. Catal..

[30] Zhang X-H (2017). Synthesis of 1, 4-diethynylbenzene-based conjugated polymer photocatalysts and their enhanced visible/near-infrared-light-driven hydrogen production activity. J. Catal..

[31] Wang K (2017). Covalent triazine frameworks via a low‐temperature polycondensation approach. Angew. Chem. Int. Ed..

[32] Byun J, Huang W, Wang D, Li R, Zhang KA (2018). CO_2_‐Triggered switchable hydrophilicity of a heterogeneous conjugated polymer photocatalyst for enhanced catalytic activity in water. Angew. Chem. Int. Ed..

[33] Jiang J-X (2008). Conjugated microporous poly(phenylene butadiynylene)s. Chem. Commun..

[34] Zhuang X (2013). Two‐dimensional sandwich‐Type, graphene‐based conjugated microporous polymers. Angew. Chem. Int. Ed..

[35] Wang L (2017). Conjugated microporous polymer nanosheets for overall water splitting using visible light. Adv. Mater..

[36] Dawson R, Adams DJ, Cooper AI (2011). Chemical tuning of CO_2_ sorption in robust nanoporous organic polymers. Chem. Sci..

[37] Ghosh S (2015). Conducting polymer nanostructures for photocatalysis under visible light. Nat. Mater..

[38] Xu Y, Chen L, Guo Z, Nagai A, Jiang D (2011). Light-emitting conjugated polymers with microporous network architecture: interweaving scaffold promotes electronic conjugation, facilitates exciton migration, and improves luminescence. J. Am. Chem. Soc..

[39] Xiang Y (2018). Conjugated polymers with sequential fluorination for enhanced photocatalytic H_2_ evolution via proton-coupled electron transfer. ACS Energy Lett..

[40] Saeki A, Seki S, Sunagawa T, Ushida K, Tagawa S (2006). Charge-carrier dynamics in polythiophene films studied by in-situ measurement of flash-photolysis time-resolved microwave conductivity (FP-TRMC) and transient optical spectroscopy (TOS). Philos. Mag..

[41] Chen L, Honsho Y, Seki S, Jiang D (2010). Light-harvesting conjugated microporous polymers: rapid and highly efficient flow of light energy with a porous polyphenylene framework as antenna. J. Am. Chem. Soc..

[42] Wang S, Guan BY, Lu Y, Lou XWD (2017). Formation of hierarchical In_2_S_3_-CdIn_2_S_4_ heterostructured nanotubes for efficient and stable visible light CO_2_ reduction. J. Am. Chem. Soc..

[43] Krishnan C, Sutin N (1981). Homogeneous catalysis of the photoreduction of water by visible light Mediation by a tris(2,2’-bipyridine) ruthenium (II)-cobalt (II) bipyridine system. J. Am. Chem. Soc..

[44] Meng X (2017). Efficient photocatalytic CO_2_ reduction in all-inorganic aqueous environment: cooperation between reaction medium and Cd (II) modified colloidal ZnS. Nano Energy.

[45] Guan X-H, Chen G-H, Shang C (2007). ATR-FTIR and XPS study on the structure of complexes formed upon the adsorption of simple organic acids on aluminum hydroxide. J. Environ. Sci..

[46] Ouyang T, Huang HH, Wang JW, Zhong DC, Lu TB (2017). A dinuclear cobalt cryptate as a homogeneous photocatalyst for highly selective and efficient visible‐light driven CO_2_ reduction to CO in CH_3_CN/H_2_O solution. Angew. Chem. Int. Ed..

[47] Liu M (2016). Enhanced electrocatalytic CO_2_ reduction via field-induced reagent concentration. Nature.

[48] Jiang J-X (2008). Synthetic control of the pore dimension and surface area in conjugated microporous polymer and copolymer networks. J. Am. Chem. Soc..

[49] Ren S-B (2018). A 1, 3-diyne-linked conjugated microporous polymer for selective CO_2_ capture. Ind. Eng. Chem. Res..

[50] Dawson R (2019). Impact of water coadsorption for carbon dioxide capture in microporous polymer sorbents. J. Am. Chem. Soc..

[51] Wang S (2017). Light-switchable oxygen vacancies in ultrafine Bi_5_O_7_Br nanotubes for boosting solar-driven nitrogen fixation in pure water. Adv. Mater..

