# Supplementary material for: Intermolecular cascaded π-conjugation channels for electron delivery powering CO2 photoreduction
Source: Nat Commun. 2020 Mar 2;11:1149. doi: 10.1038/s41467-020-14851-7 (PMC7051963; doi:10.1038/s41467-020-14851-7)
Supplement: Supplementary file 1 — Supplementary Information [file 41467_2020_14851_MOESM1_ESM.pdf]

Wang *et al.*

Supplementary Information

Intermolecular Cascaded π-Conjugation Channels for Electron Delivery Powering CO_2 Photoreduction

Shengyao Wang[1,2], Xiao Hai[2,3], Xing Ding[1], Shangbin Jin[4], Yonggang Xiang[1], Pei Wang[1], Bo Jiang[2], Fumihiko Ichihara[2,3], Mitsutake Oshikiri[2], Xianguang Meng[2], Yunxiang Li[2,3], Wakana Matsuda[5], Jun Ma[5], Shu Seki[5], Xuepeng Wang[1], Hao Huang[2], Yoshiki Wada[6], Hao Chen*[1] and Jinhua Ye*[2,3,7]

[1]College of Science, Huazhong Agricultural University, Wuhan 430070, P. R. China.

[2]International Center for Materials Nanoarchitectonics (WPI-MANA), National Institute for Materials Science (NIMS), 1-1 Namiki, Tsukuba, Ibaraki 305-0055, Japan.

[3]Graduates School of Chemical Science and Engineering, Hokkaido University, Sapporo 060-0814, Japan.

[4]Key Laboratory of Material Chemistry for Energy Conversion and Storage, Ministry of Education, School of Chemistry and Chemical Engineering, Huazhong University of Science and Technology, Wuhan 430074, P. R. China.

[5]Department of Molecular Engineering, Kyoto University, Kyoto 615-8510, Japan.

[6]Electroceramics Group, National Institute for Materials Science (NIMS), 1-1 Namiki, Tsukuba, Ibaraki 305-0055, Japan.

[7]TU-NIMS Joint Research Center, School of Materials Science and Engineering, Tianjin 300072, P. R. China.

Supplementary Figures

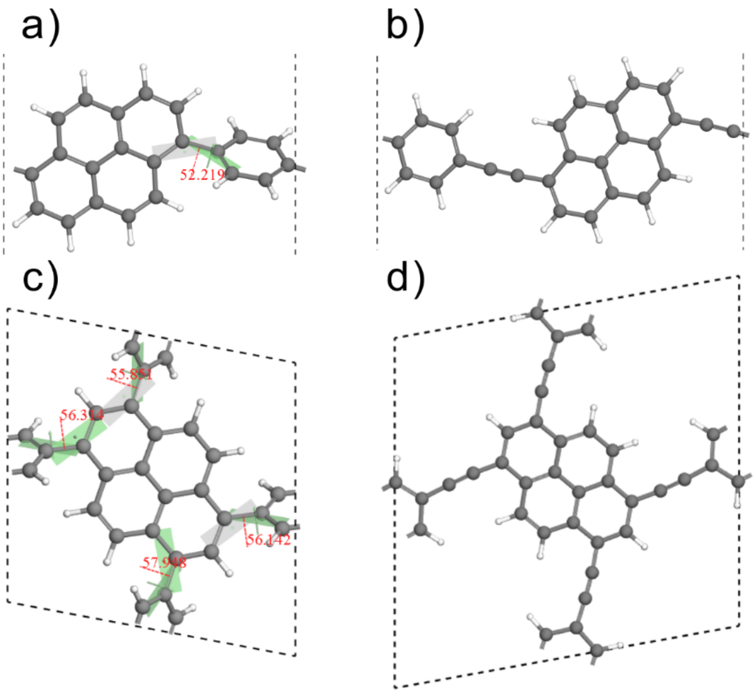

Supplementary Figure 1. Chemical Structures of L-CP-D (a), L-CP-A (b), N-CP-D (c) and N-CP-A (d). The dark grey and white balls represent C and H atoms, respectively. The dihedral angle was listed (red) for L-CP-D and N-CP-D, while the grey and light green plane represented the corresponding plane.

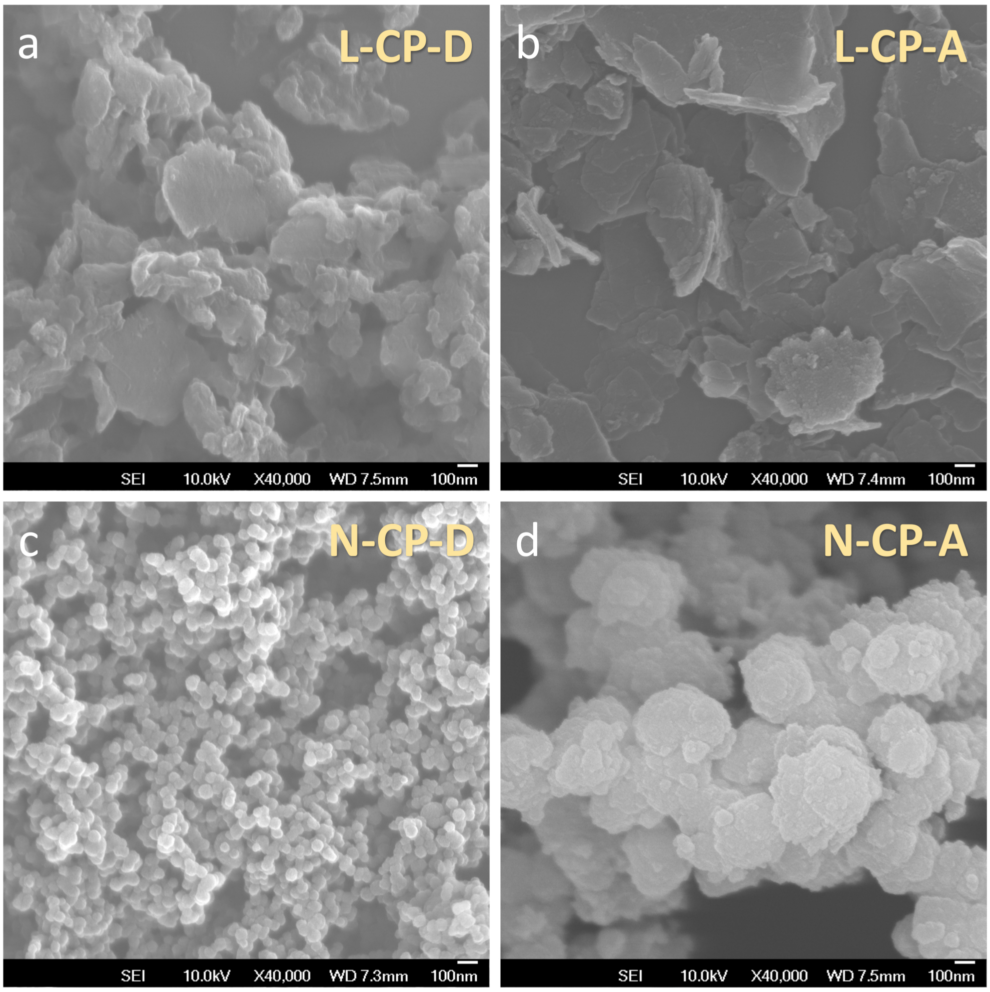

Supplementary Figure 2. Scanning electron microscopy images of L-CP-D (a), L-CP-A (b), N-CP-D (c) and N-CP-A (d).

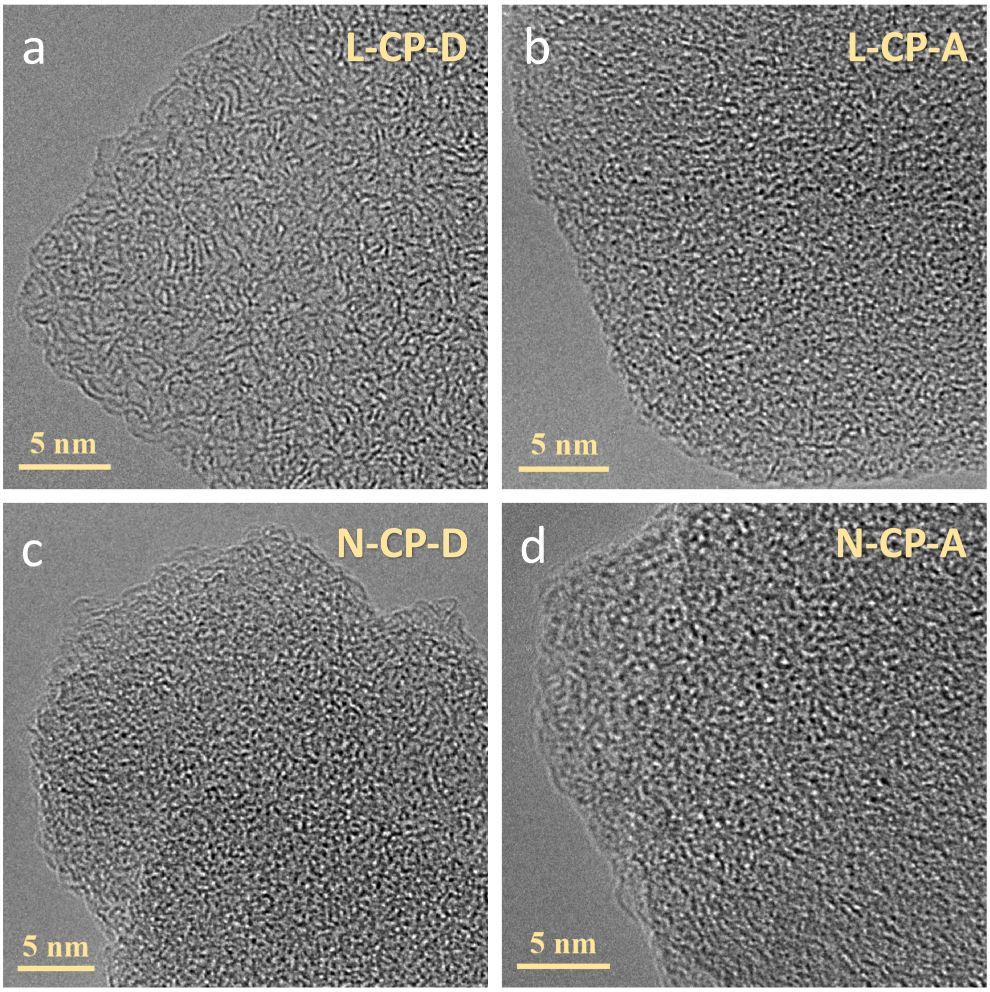

Supplementary Figure 3. Transmission electron microscopy images of L-CP-D (a), L-CP-A (b), N-CP-D (c) and N-CP-A (d).

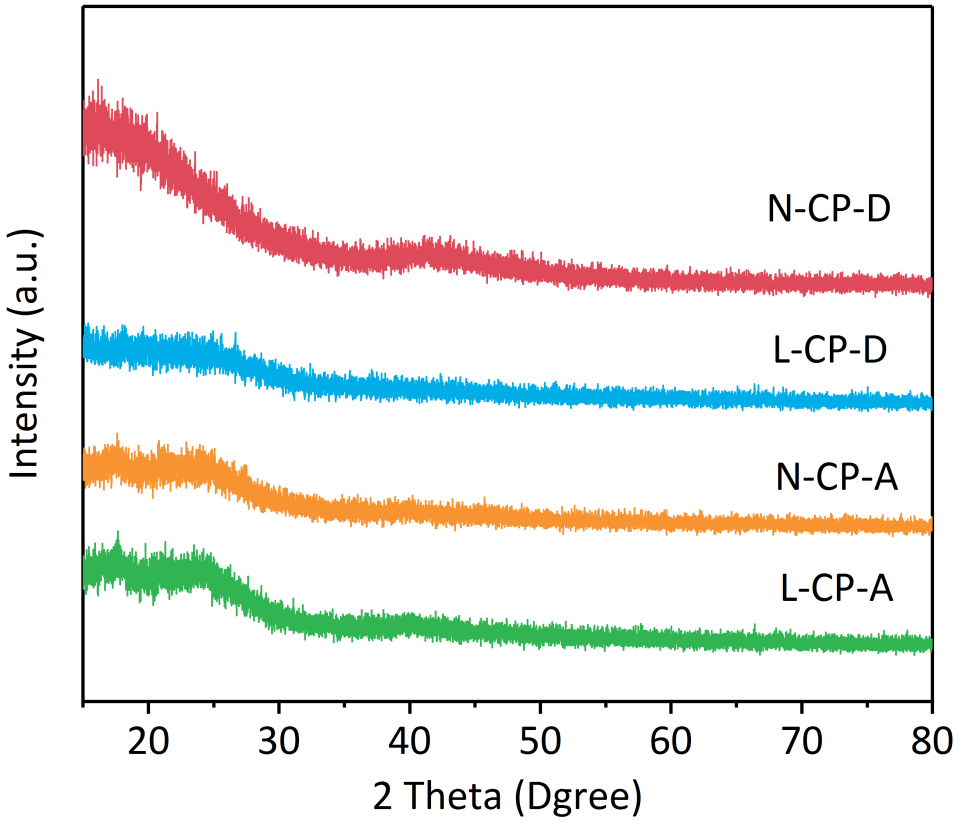

Supplementary Figure 4. Powder X-ray diffraction patterns of L-CP-D, L-CP-A, N-CP-D and N-CP-A.

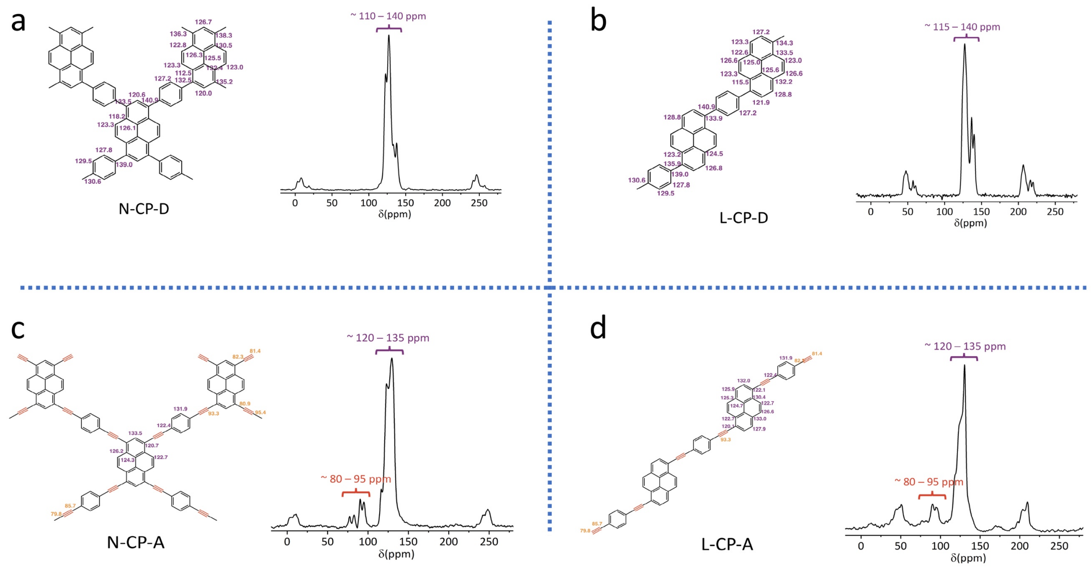

Supplementary Figure 5. Estimated chemical shifts of N-CP-D (a), L-CP-D (b), N-CP-A (c) and L-CP-A (d) and the corresponding solid-state ^{13}C cross-polarization/magic angle spinning nuclear magnetic resonance.

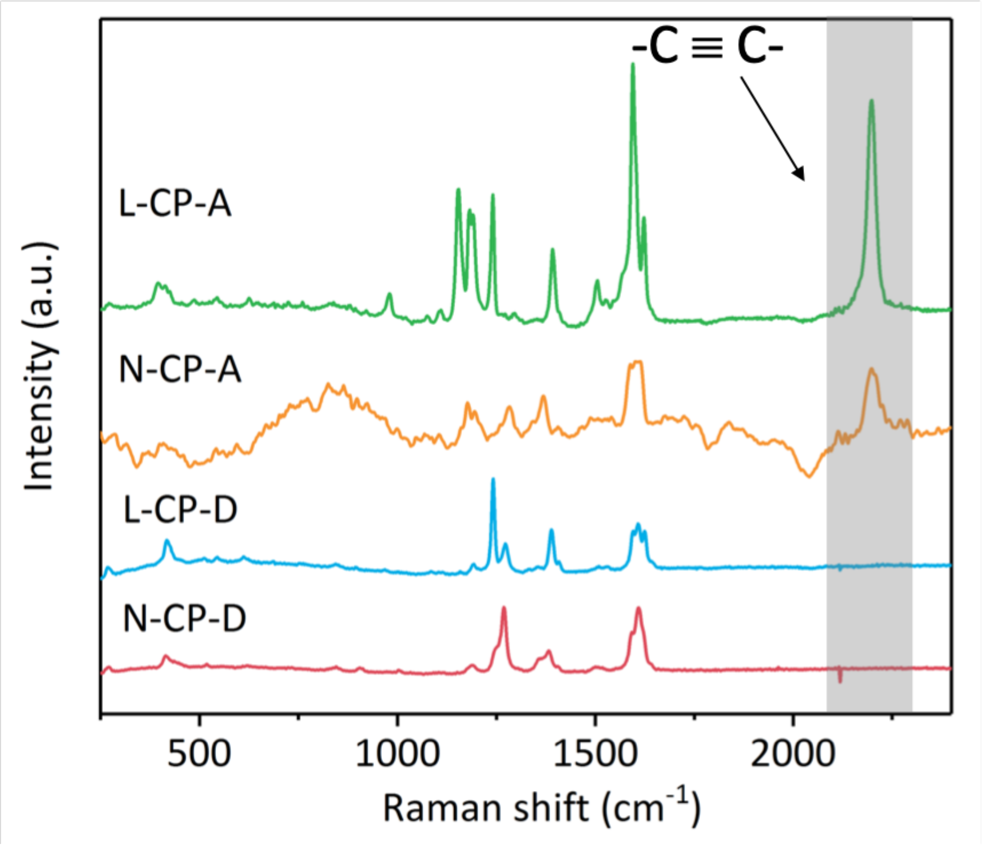

Supplementary Figure 6. Raman spectroscopy of L-CP-D, L-CP-A, N-CP-D and N-CP-A.

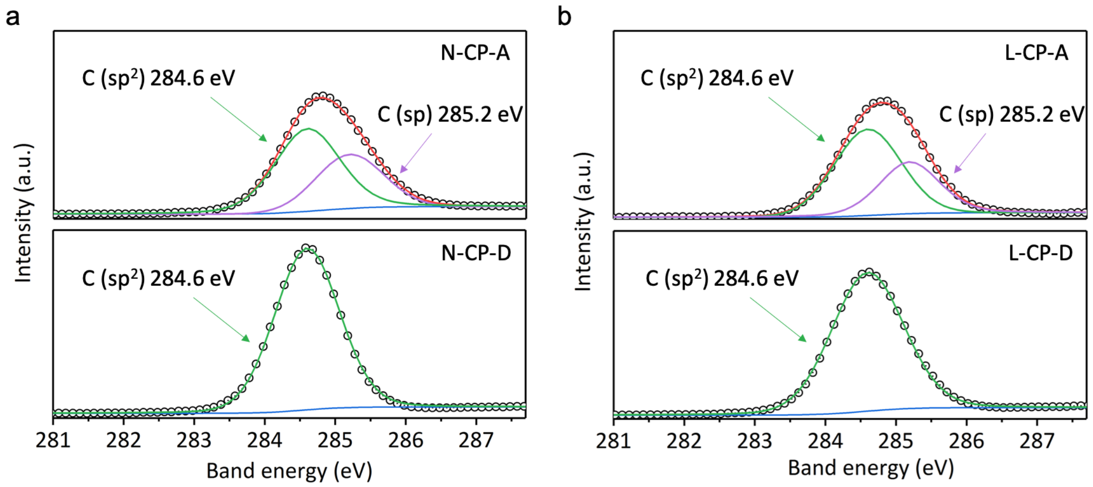

Supplementary Figure 7. X-ray photoelectron spectroscopy of N-CP-A and N-CP-D (a), L-CP-A and L-CP-D (b).

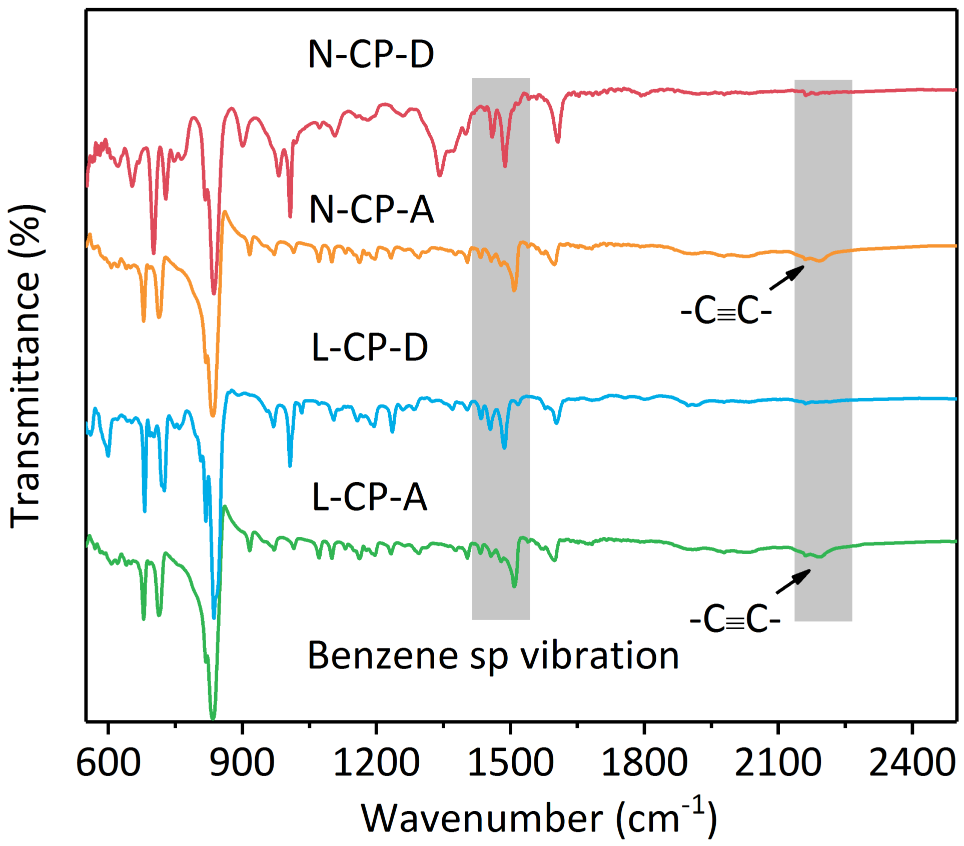

Supplementary Figure 8. Fourier transform infrared spectroscopy of L-CP-D, L-CP-A, N-CP-D and N-CP-A.

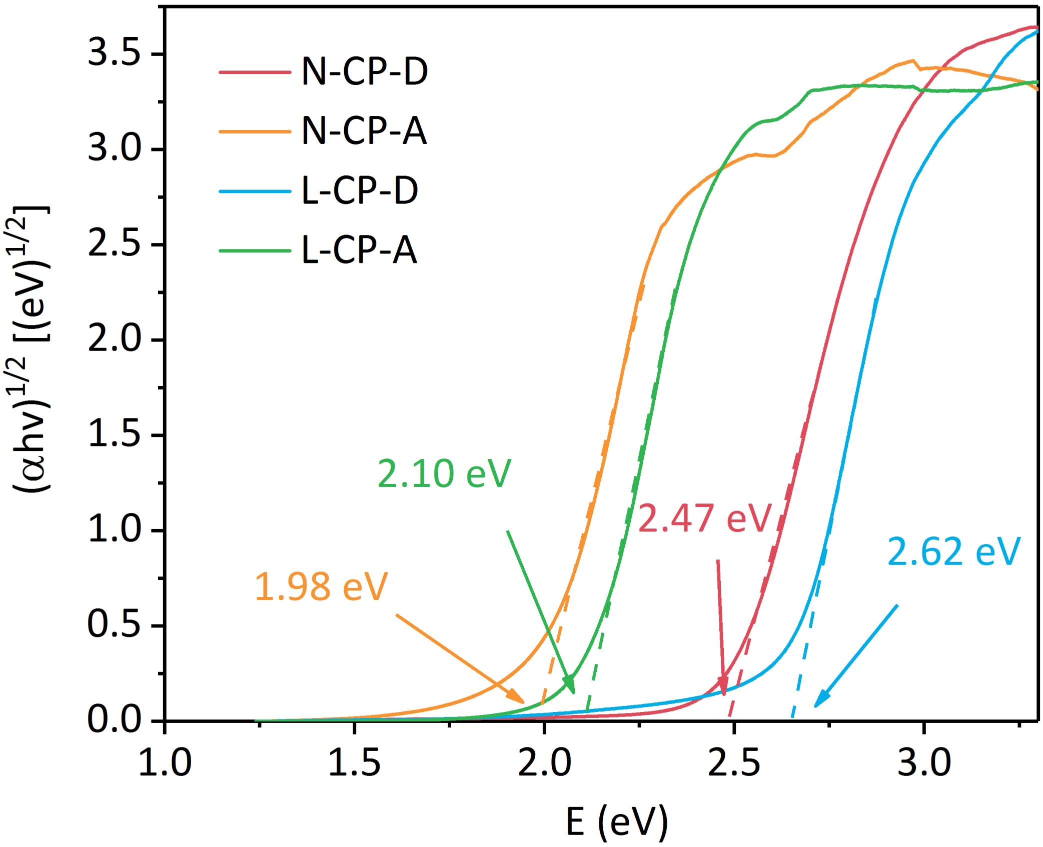

Supplementary Figure 9. Bandgap energies of L-CP-D, L-CP-A, N-CP-D and N-CP-A according to the Kubelka-Munk Function.

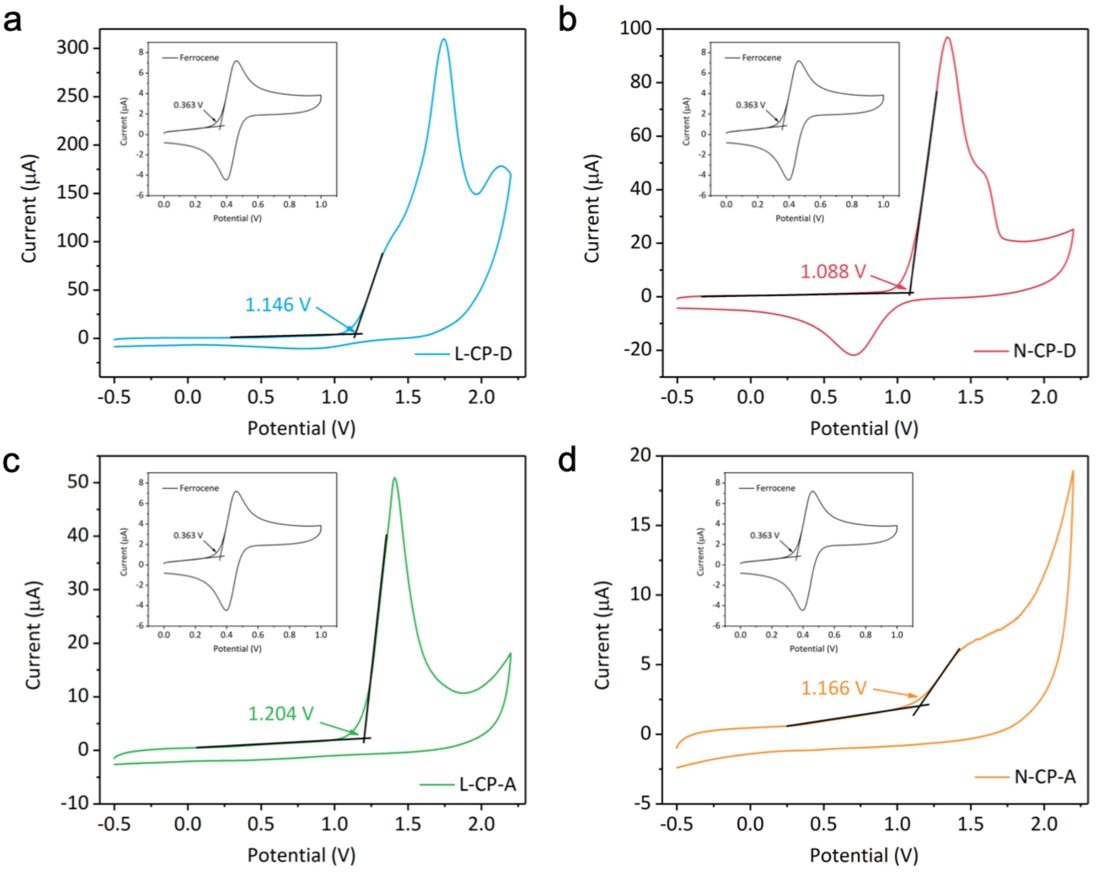

Supplementary Figure 10. Cyclic voltammograms of L-CP-D (a), N-CP-D (b), L-CP-A (c) and N-CP-A (d), Inset: the ferrocene/ferrocenium (Fc/Fc^{+}) couple was provided for an internal reference.

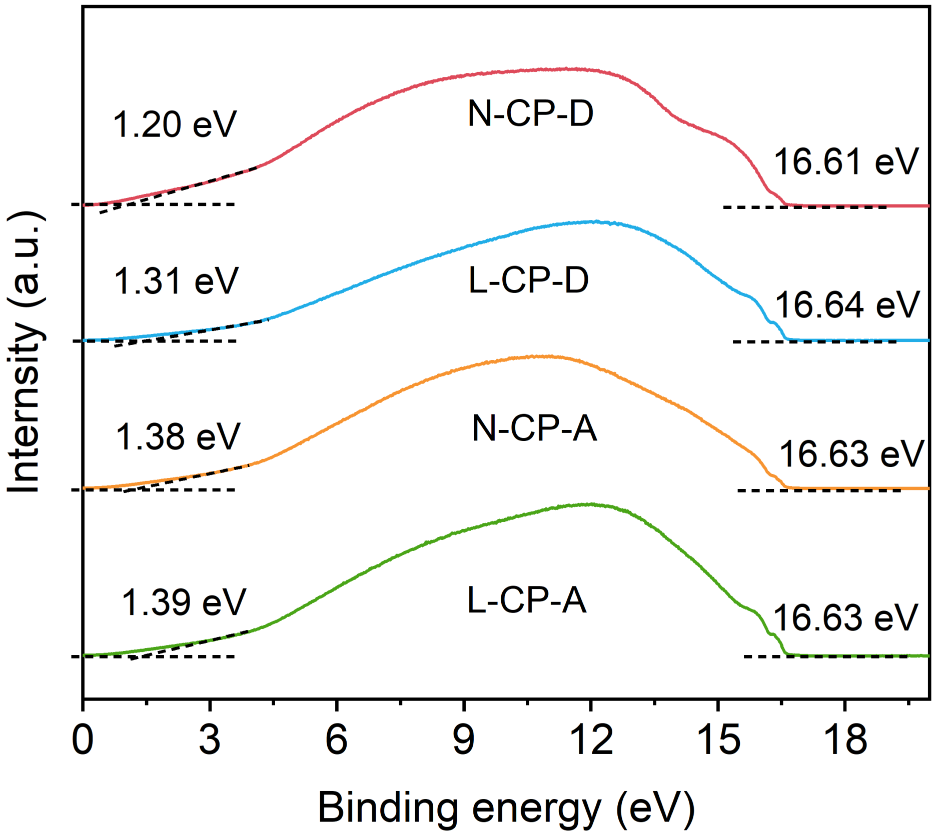

Supplementary Figure 11. **UPS spectra** in the cutoff and the onset energy regions of L-CP-D, L-CP-A, N-CP-D and N-CP-A.

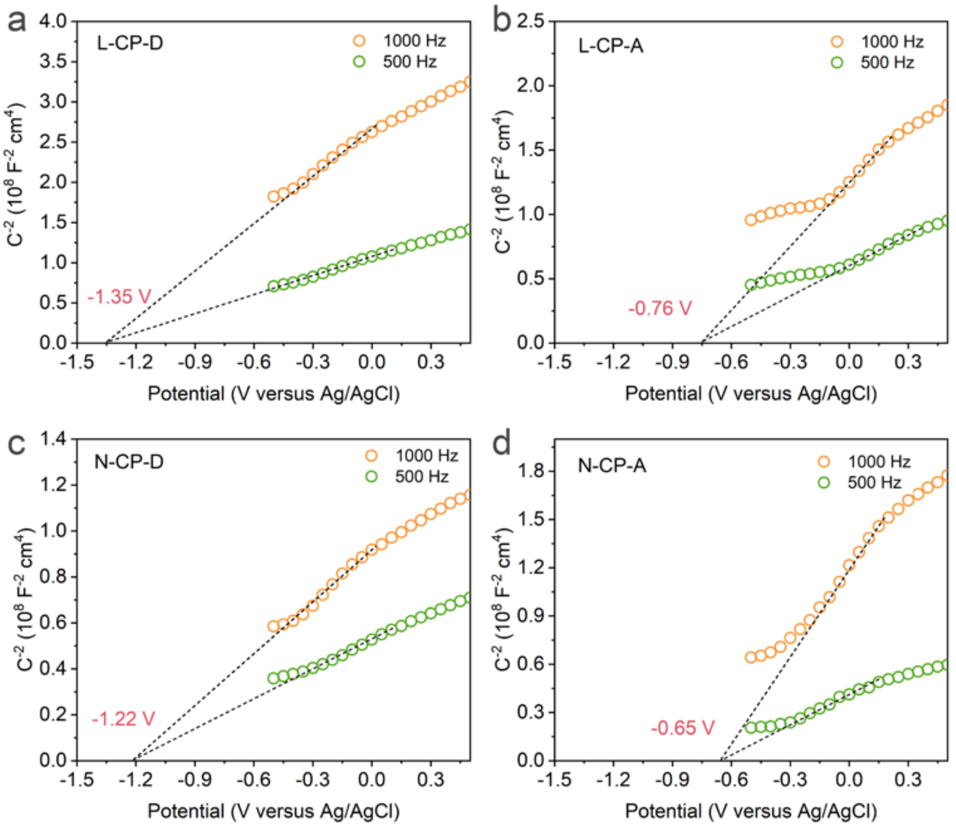

Supplementary Figure 12. **Mott-Schottky plots** for L-CP-D (a), L-CP-A (b), N-CP-D (c) and N-CP-A (d) in 0.1 M Na_2SO_4 at pH=7 by using Ag/AgCl with saturated KCl as reference electrode.

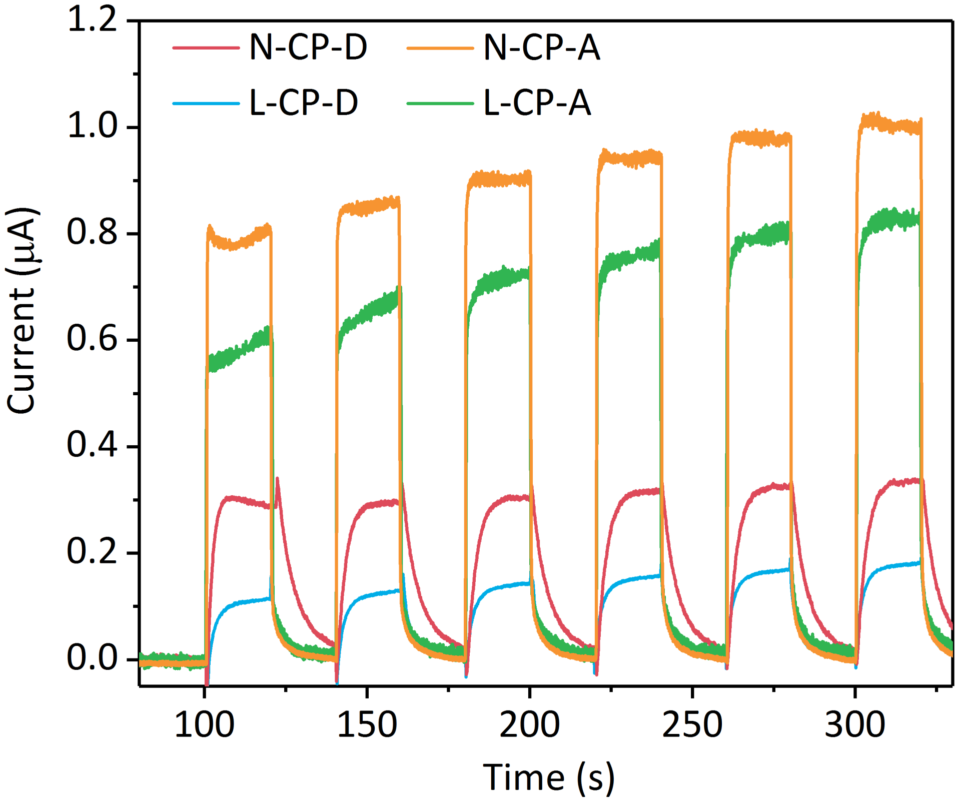

Supplementary Figure 13. Photocurrent measurement of L-CP-D, L-CP-A, N-CP-D and N-CP-A.

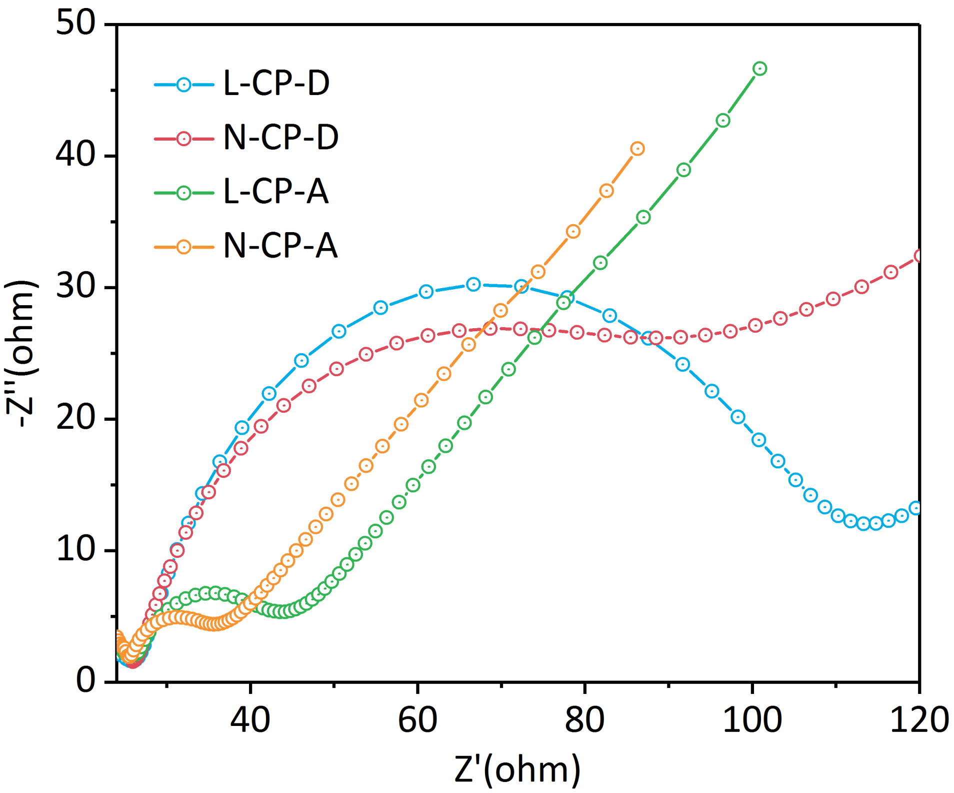

Supplementary Figure 14. Electrochemical impedance spectroscopy Nyquist plots of L-CP-D, L-CP-A, N-CP-D and N-CP-A.

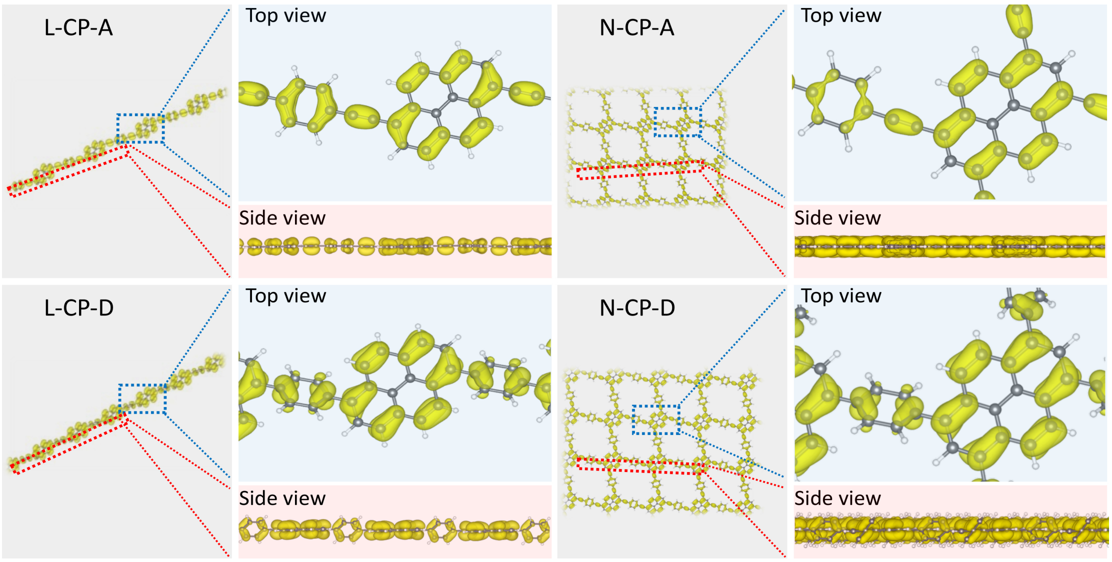

Supplementary Figure 15. The views on the charge distribution of HOMO at the Γk-point of L-CP-D, L-CP-A, N-CP-D and N-CP-A.

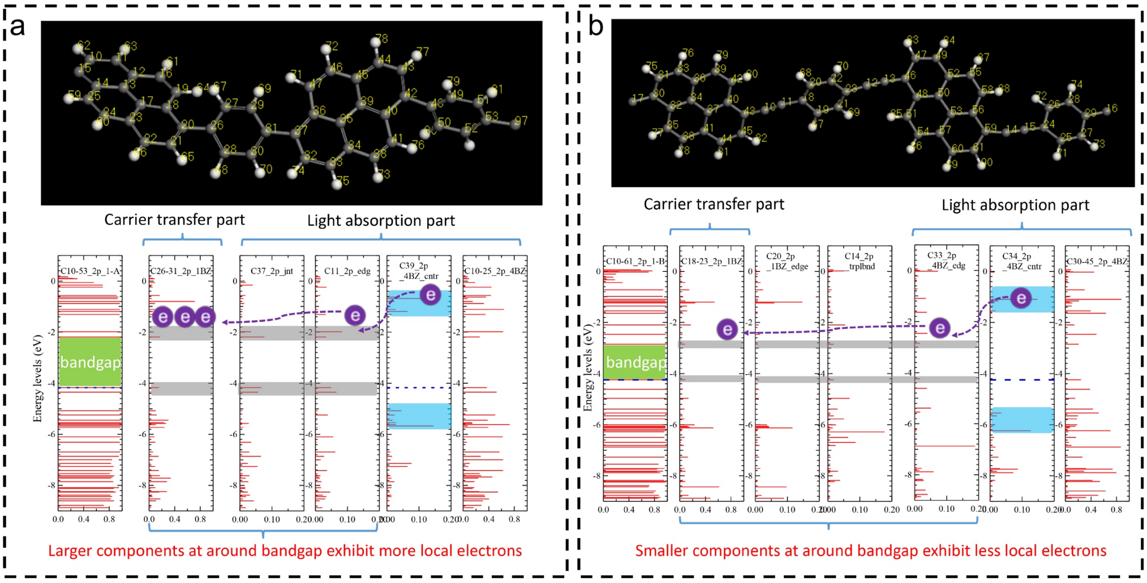

Supplementary Figure 16. Electronic comparison of backbone architecture between without (a) and with (b) alkynyl based on Becke-Lee-Yang-Parr (BLYP) functionals.

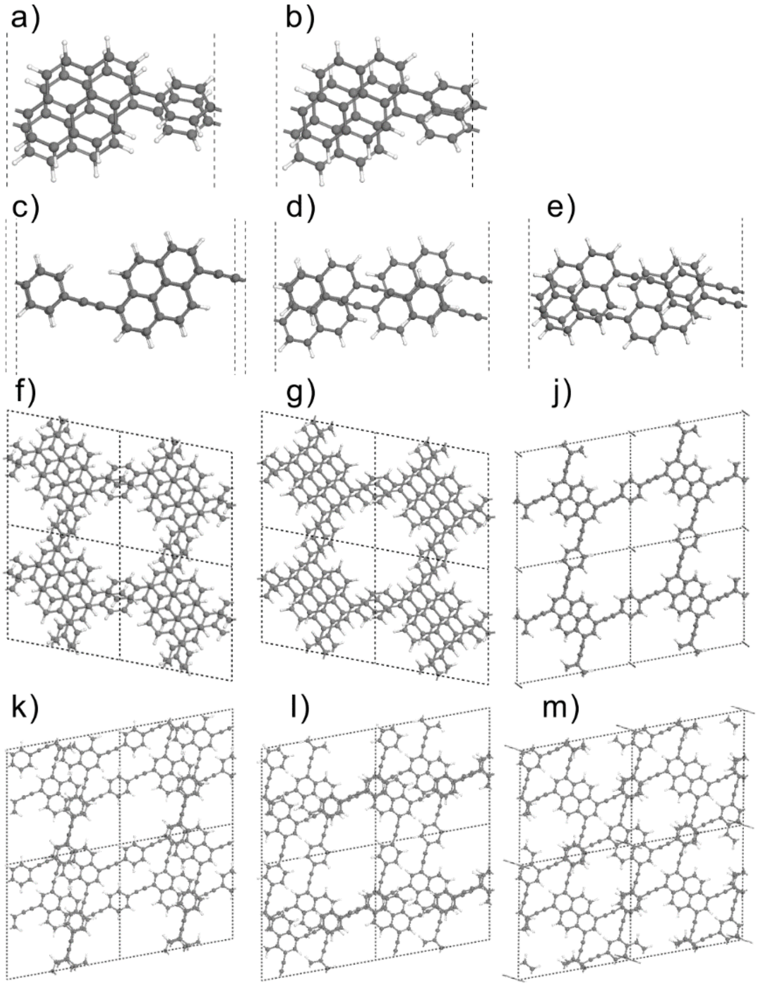

Supplementary Figure 17. The view of optimized bilayer structures of L-CP-D with (a) AB stacking and (b) AB’ stacking, L-CP-A with (c) AA stacking, (d) AB stacking and (e)AB’ stacking, N-CP-D with (f) AB stacking and (g) AB’ stacking, and N-CP-A with (j) AA stacking, (k) AB stacking and (l) AB’ stacking and (m) AC stacking. The dark gray and white balls represent C and H atoms, respectively.

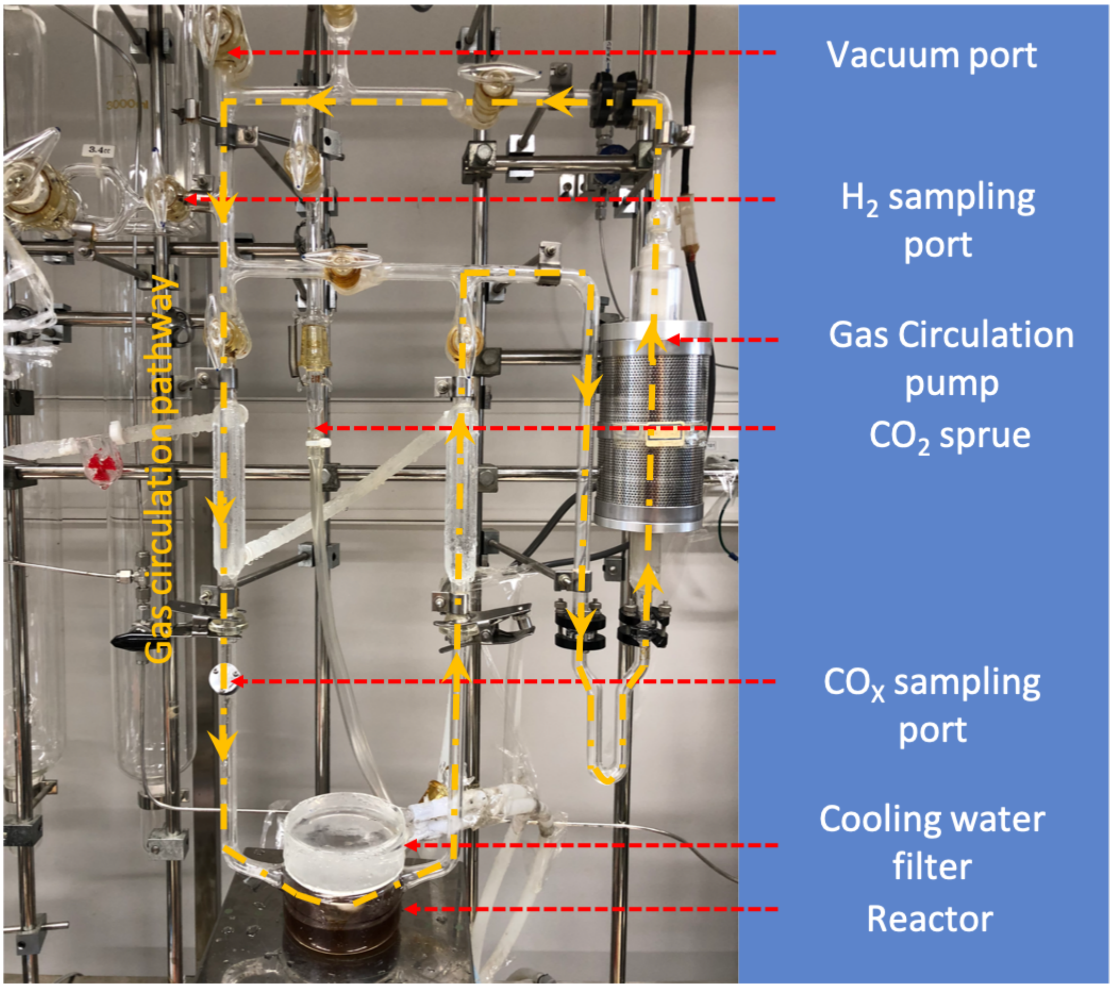

Supplementary Figure 18. The closed gas circulation system for visible light photocatalytic CO_2 reduction.

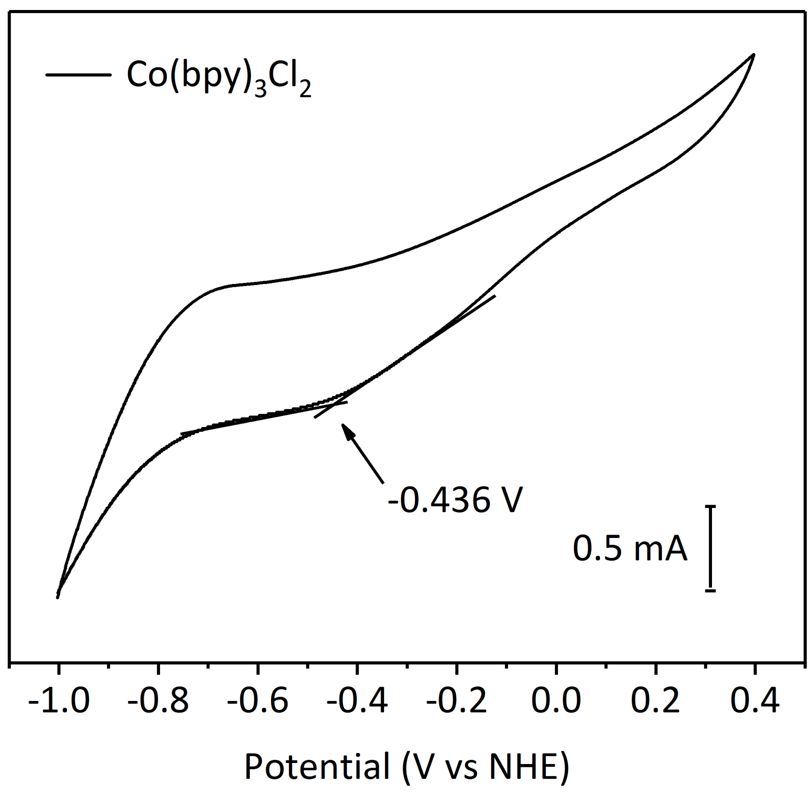

Supplementary Figure 19. Cyclic voltammograms of Co (II) bipyridine complexes in mixture of acetonitrile/water (7:3) and the redox potential can be assigned to the recycle of Co (II)/Co (I).

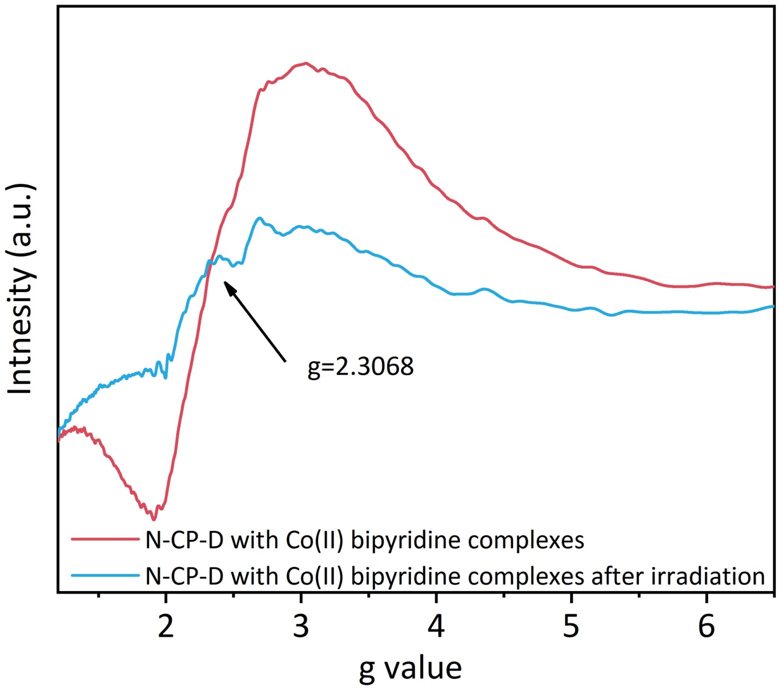

Supplementary Figure 20. Electron spin resonance spectra of N-CP-D with Co (II) bipyridine complexes under irradiation and dark condition.

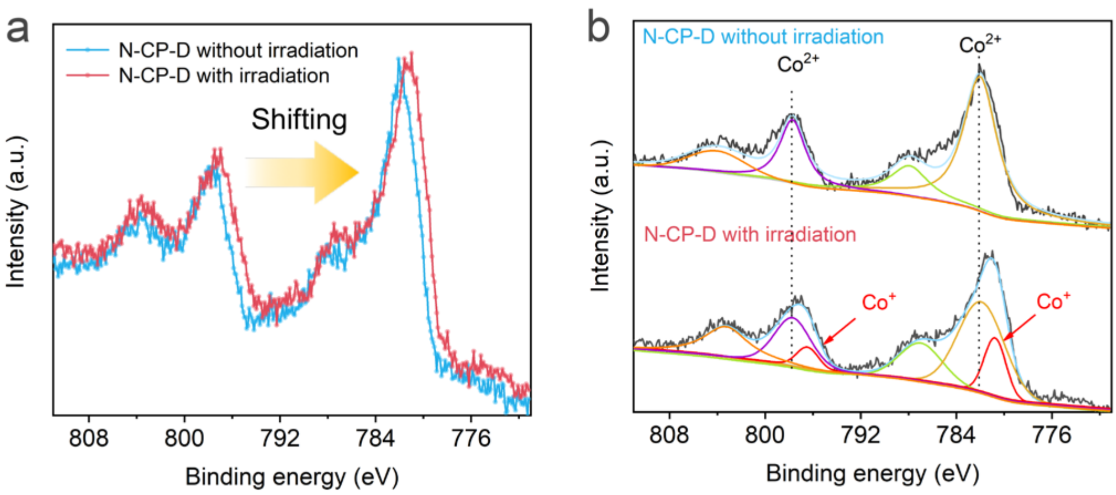

Supplementary Figure 21. In-situ XPS spectra of N-CP-D with Co complexes (a) and the corresponding multi peaks separation spectra (b).

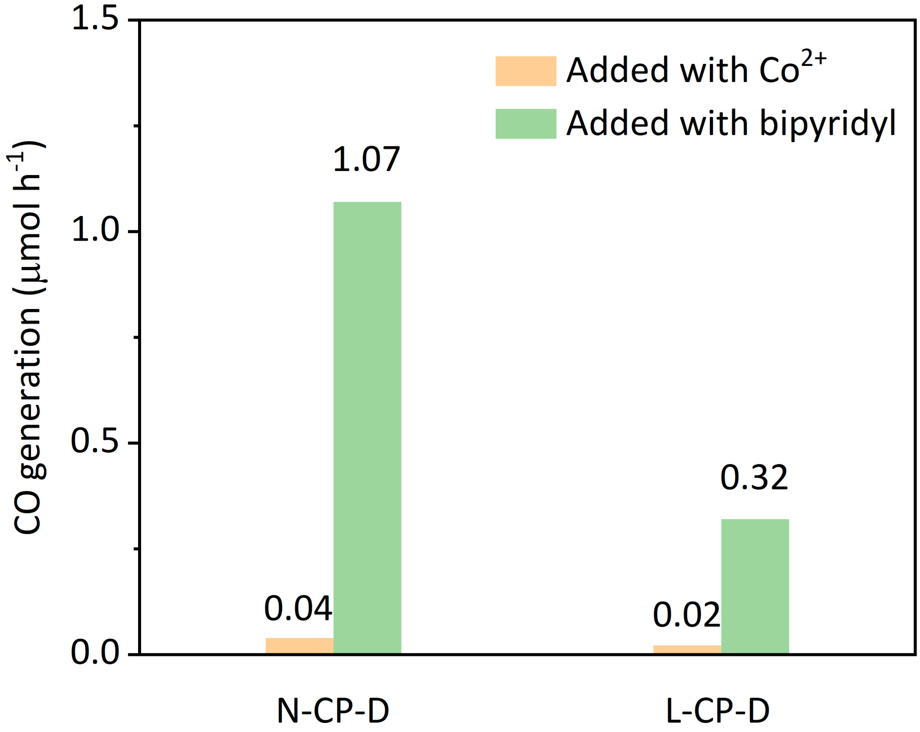

Supplementary Figure 22. Photocatalytic CO generation of N-CP-D or L-CP-D under the condition of using the same 5μmol of isolated cobalt chloride or dipyridyl as the cocatalyst.

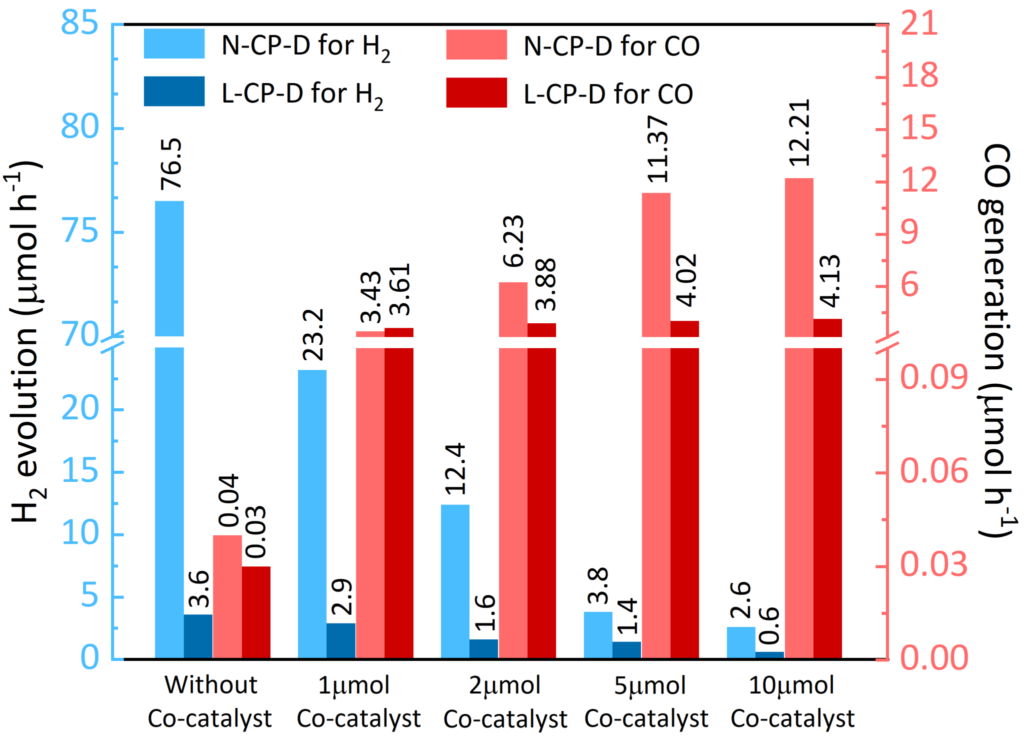

Supplementary Figure 23. The selectivity of photocatalysis over the amount of cocatalyst to the catalytic products of CO (red bars) and H_2 (blue bars) formed over L-CP-D (dark bars) and N-CP-D (light bars).

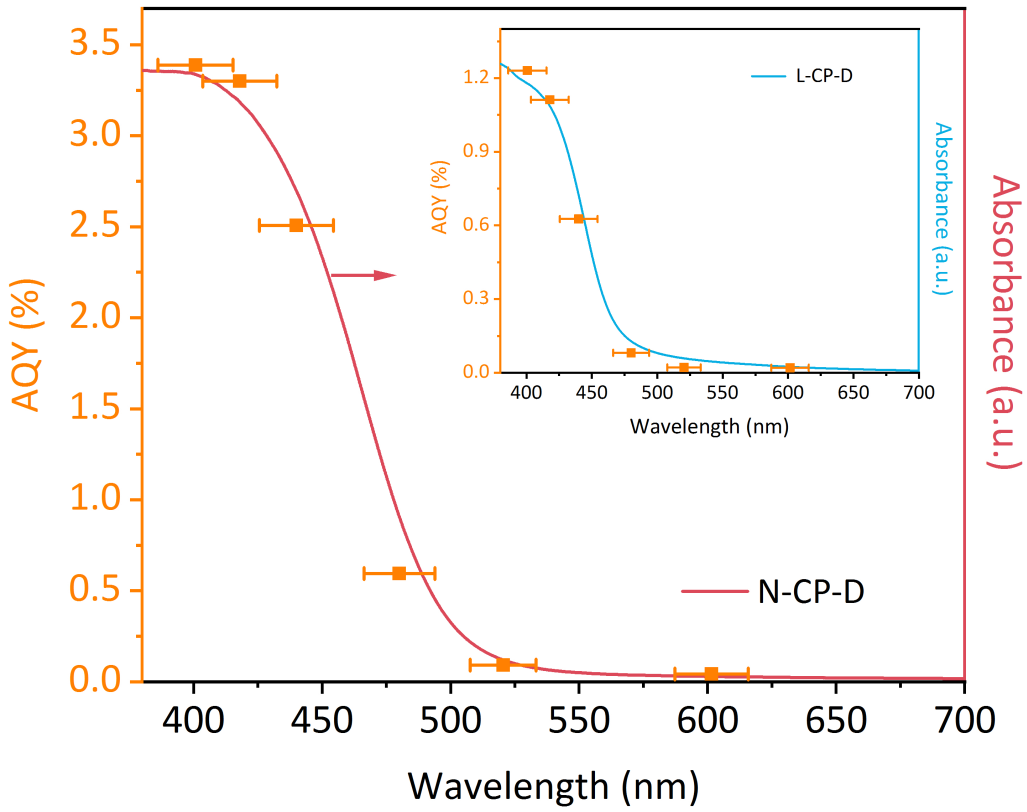

Supplementary Figure 24. Wavelength-dependent apparent quantum yield of CO generation from L-CP-D (inset) and N-CP-D.

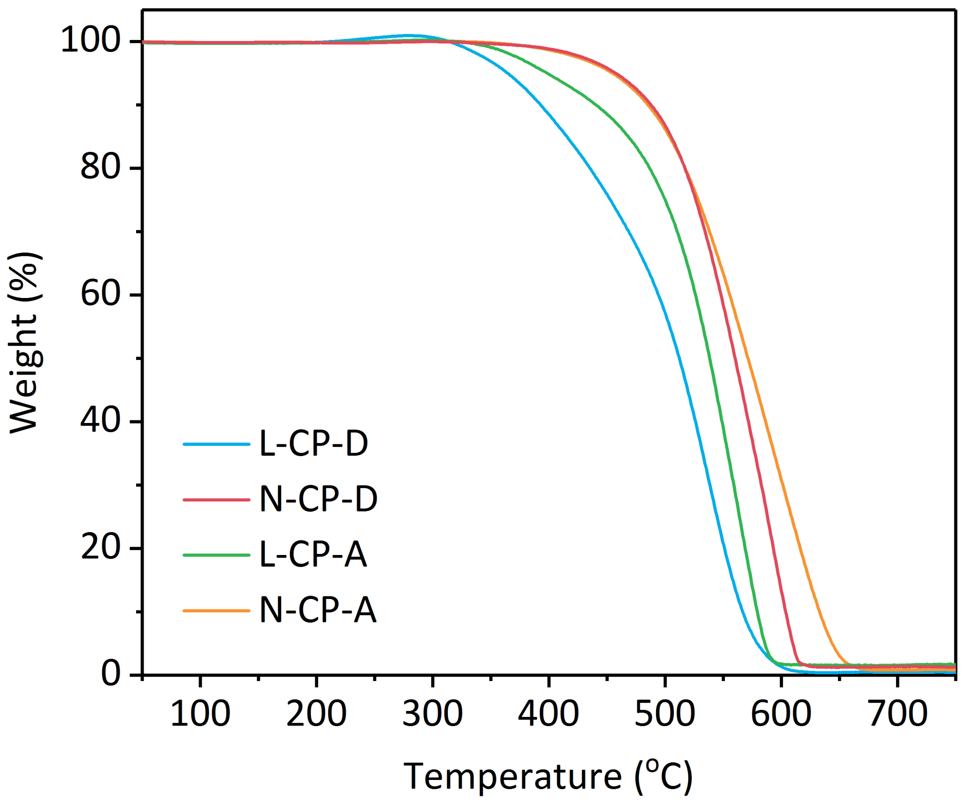

Supplementary Figure 25. Thermal gravimetric of L-CP-D, L-CP-A, N-CP-D and N-CP-A.

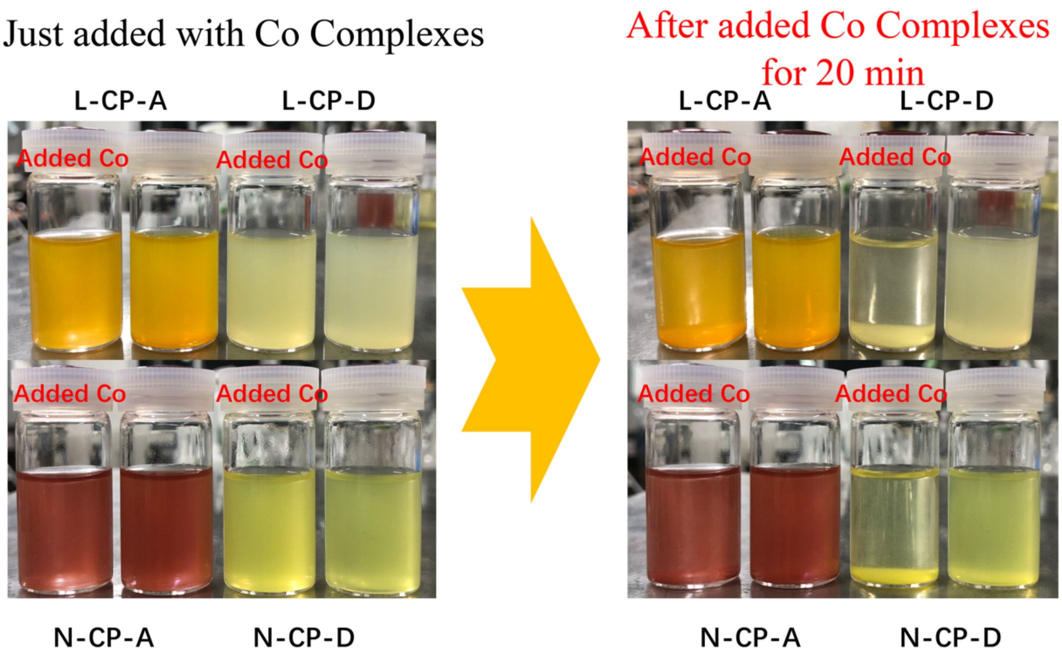

Supplementary Figure 26. The dispersion of CPs in the solvent of acetonitrile/water (7:3) mixture with or without adding Co complexes at different times.

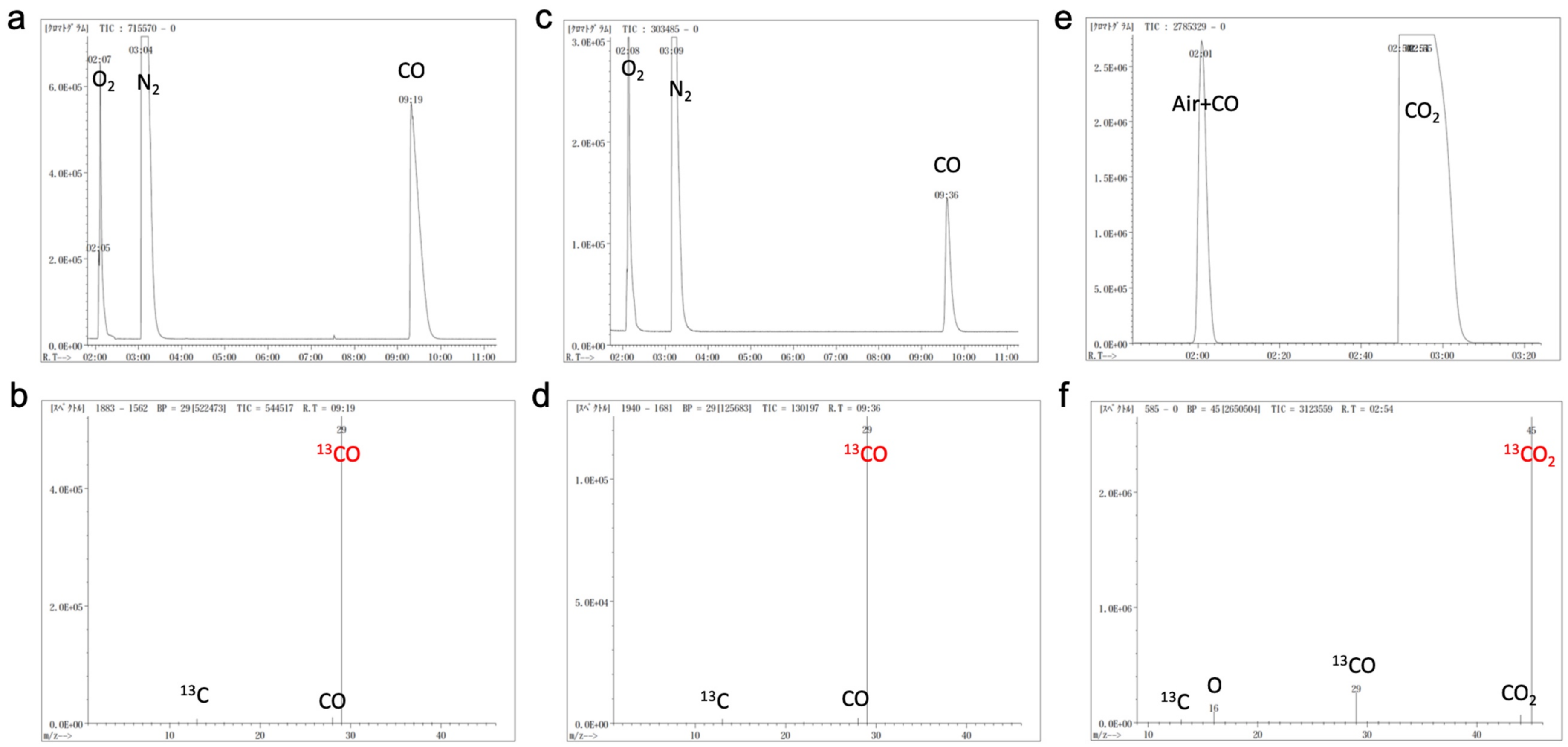

Supplementary Figure 27. The total ion chromatograph and mass spectra for the sample of N-CP-D (a, b) and L-CP-D (c, d) by using the column of HP-MOLESIEVE and total ion chromatograph and mass spectra for the sample of N-CP-D (e, f) by using the column of HP-PLOT/Q.

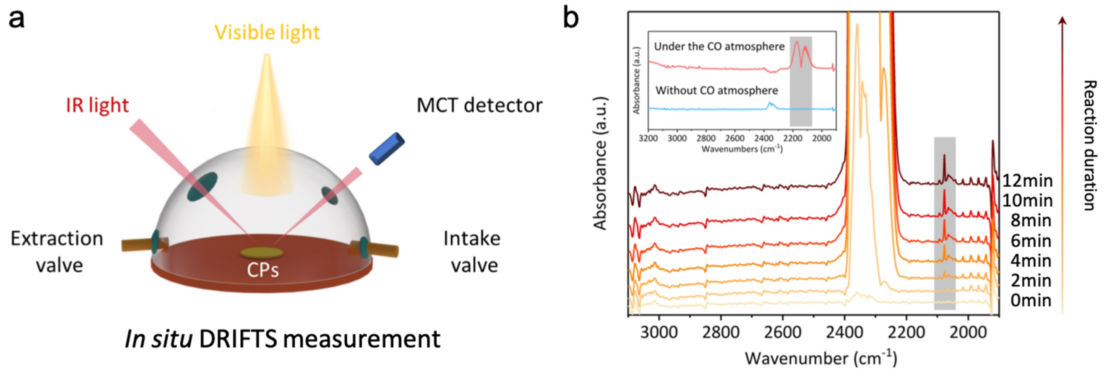

Supplementary Figure 28. In situ diffuse reflectance infrared Fourier transform spectroscopy of designed reaction cell (a) and the CO_2 photoreduction process over N-CP-D according to the DRIFTS measurement (b) inset: N-CP-D with (red line) or without (blue line) absorbed CO in DRIFTS.

.

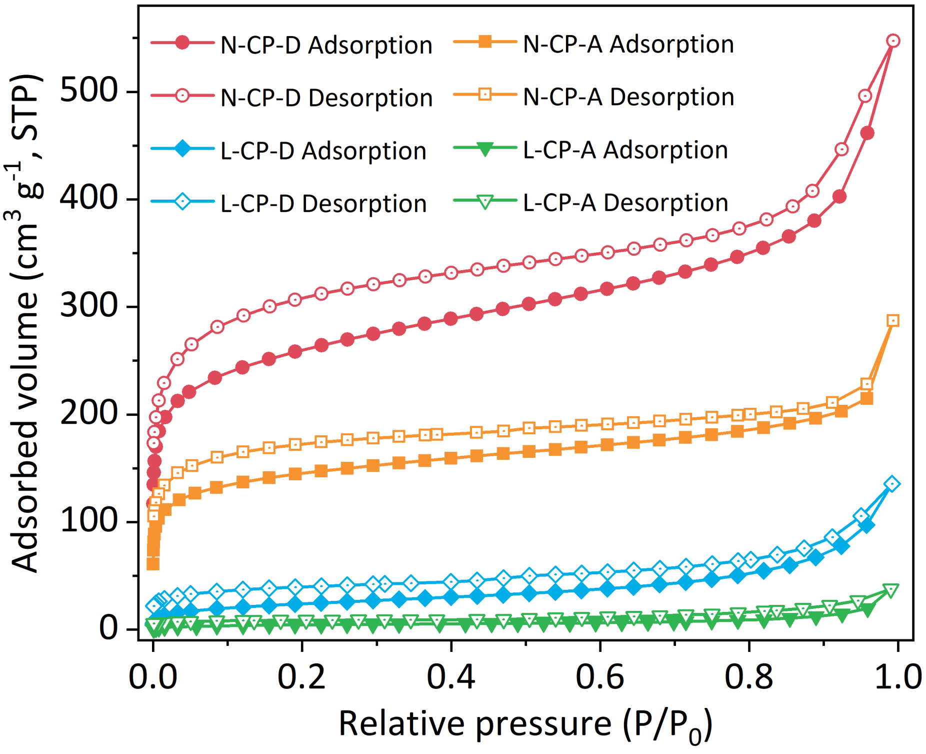

Supplementary Figure 29. Nitrogen adsorption and desorption isotherms of L-CP-D, L-CP-A, N-CP-D and N-CP-A at 77.3 K (filled symbols: adsorption, open symbols: desorption, STP: standard temperature pressure).

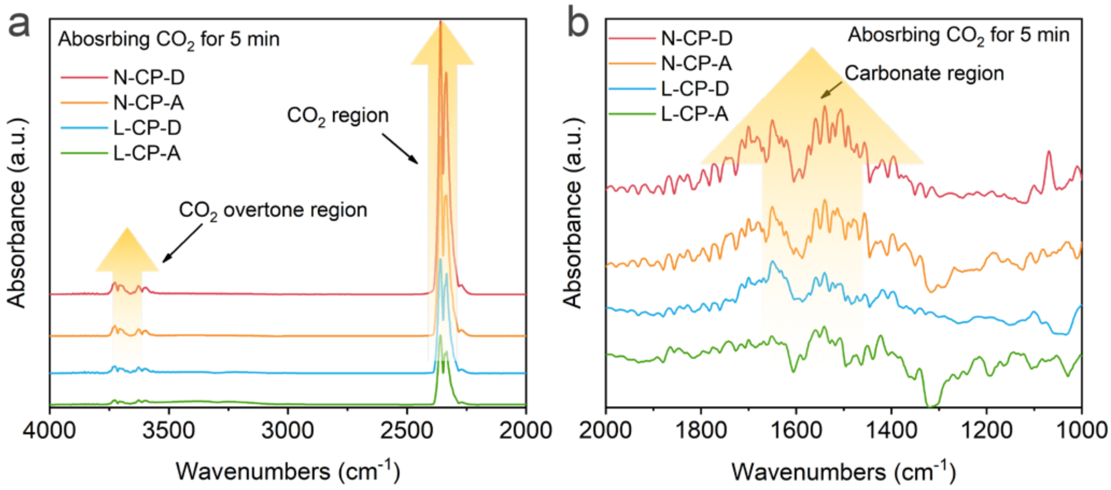

Supplementary Figure 30. In-situ FT-IR spectra of N-CP-D with Co complexes for the adsorption of CO_2 (a) and the chemisorption of CO_2 (b) in a solvent-containing environment.

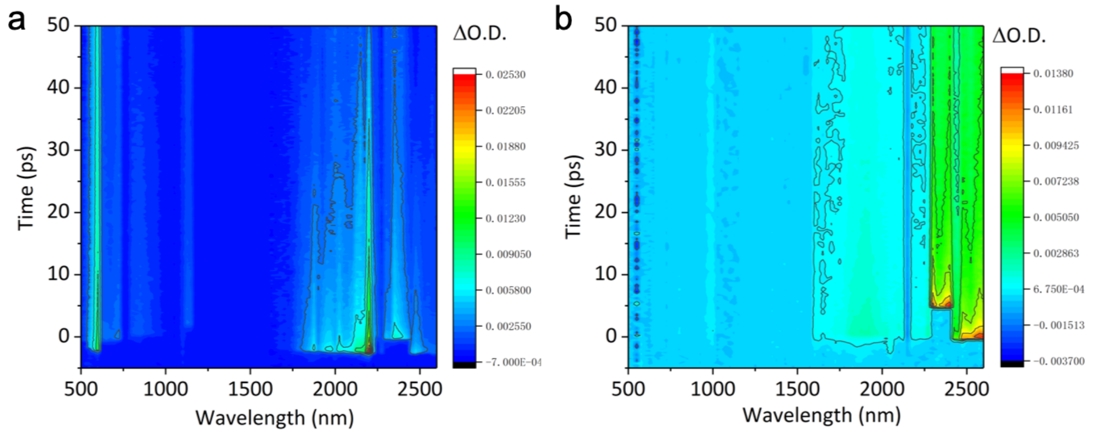

Supplementary Figure 31. Two-dimensional pseudo-color plot of TA spectra for N-CP-D (a) and N-CP-A (b).

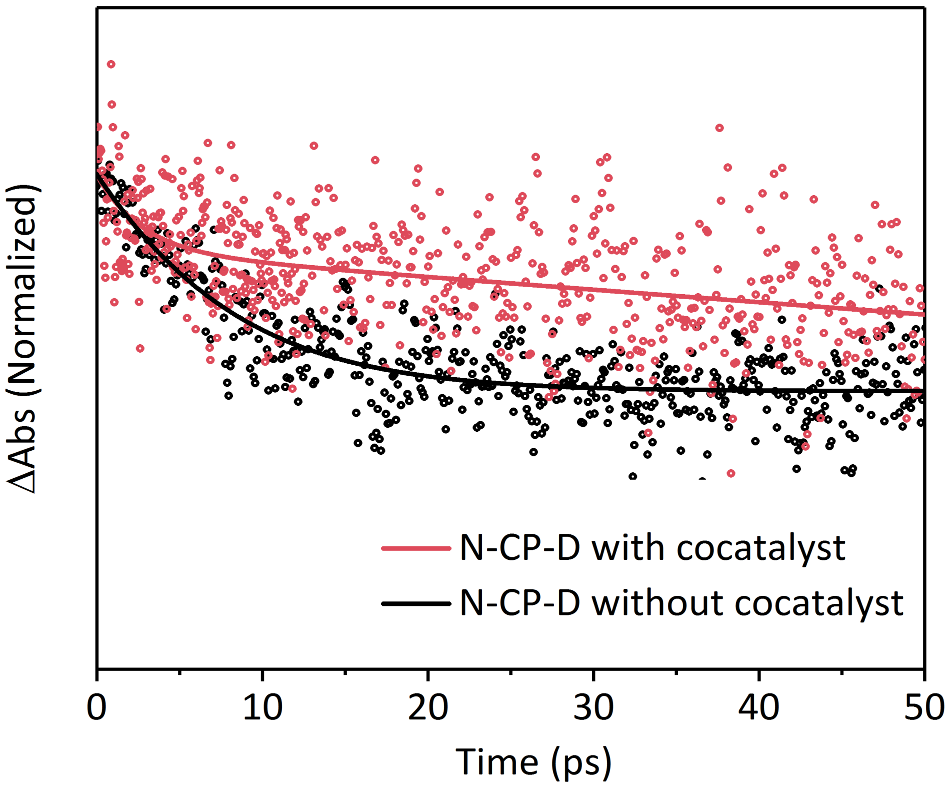

Supplementary Figure 32. Kinetics of electrons in transient absorption over N-CP-D with or without cocatalyst under a probe wavelength of 525 nm.

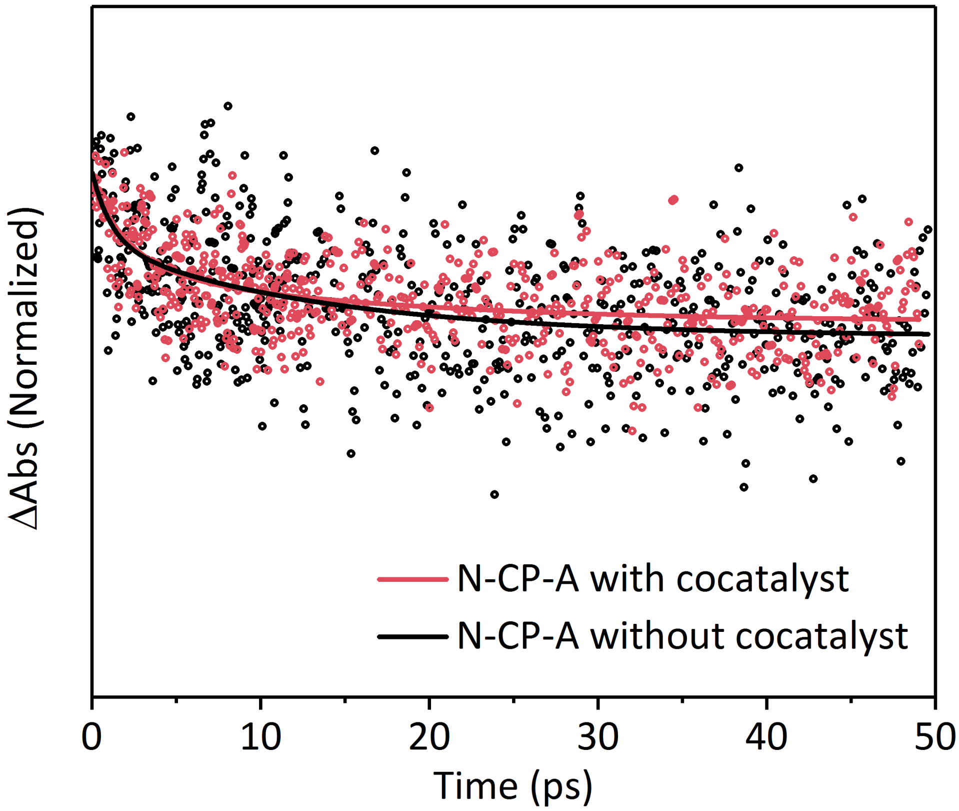

Supplementary Figure 33. Kinetics of electrons in transient absorption over N-CP-A with or without cocatalyst under a probe wavelength of 550 nm.

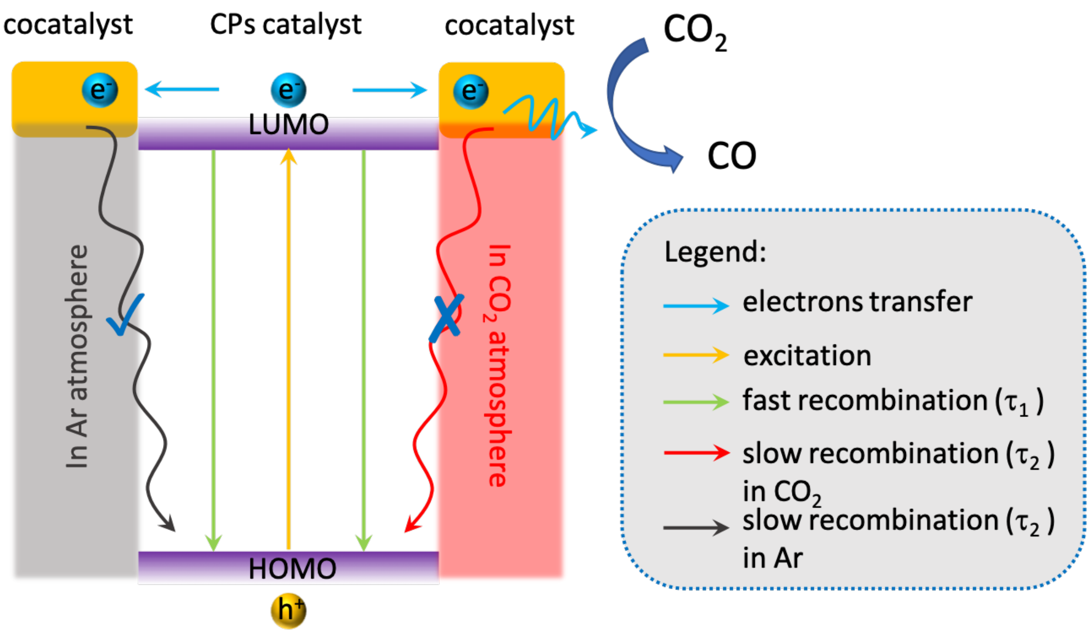

Supplementary Figure 34. The difference in time-resolved photoluminescence under CO_2 or Ar atmosphere over CPs-D series.

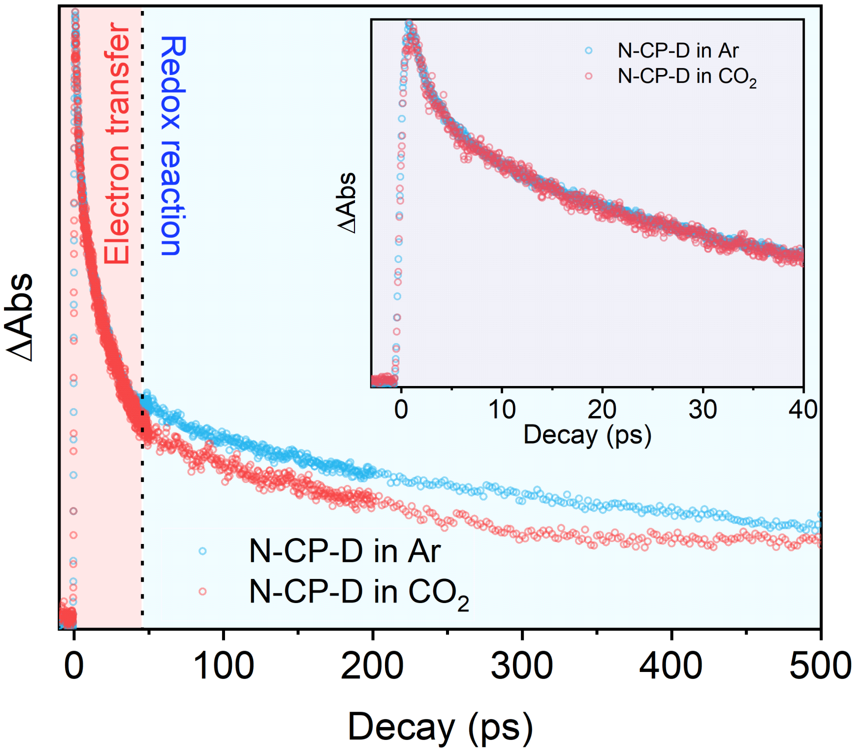

Supplementary Figure 35. Kinetics of electrons in transient absorption over N-CP-D in the presence of scavenger under the atmosphere of CO_2 or argon with a probe wavelength of 2200 nm. Inset: enlarged kinetics of electrons in first 40 ps.

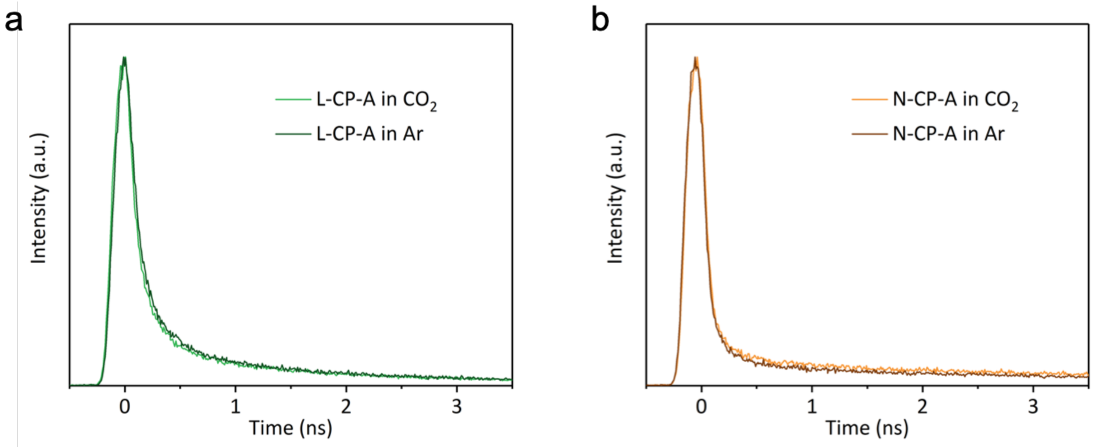

Supplementary Figure 36. **Time-resolved photoluminescence decay** of L-CP-A (a) and N-CP-A (b) under a CO_2 or argon atmosphere.

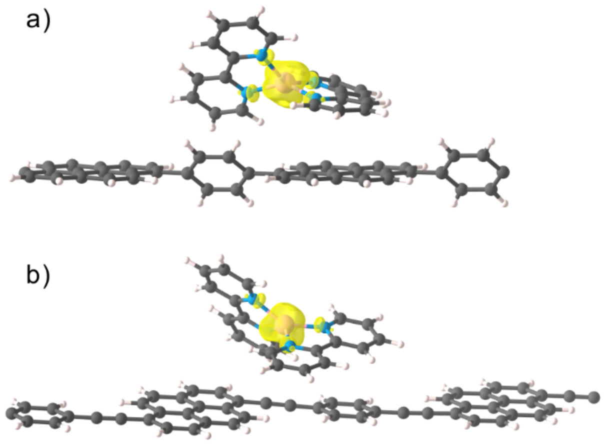

Supplementary Figure 37. Spin density of (a) $Co(bpy)_2^{2+}$/L-CP-D and (b) $Co(bpy)_2^{2+}$/L-CP-A, with isosurfaces value of 0.005 e Å^{-3}. yellow and cyan represent spin up and spin down, respectively. The pink, indigo, dark gray and white pink balls represent Co, N, C and H atoms, respectively.

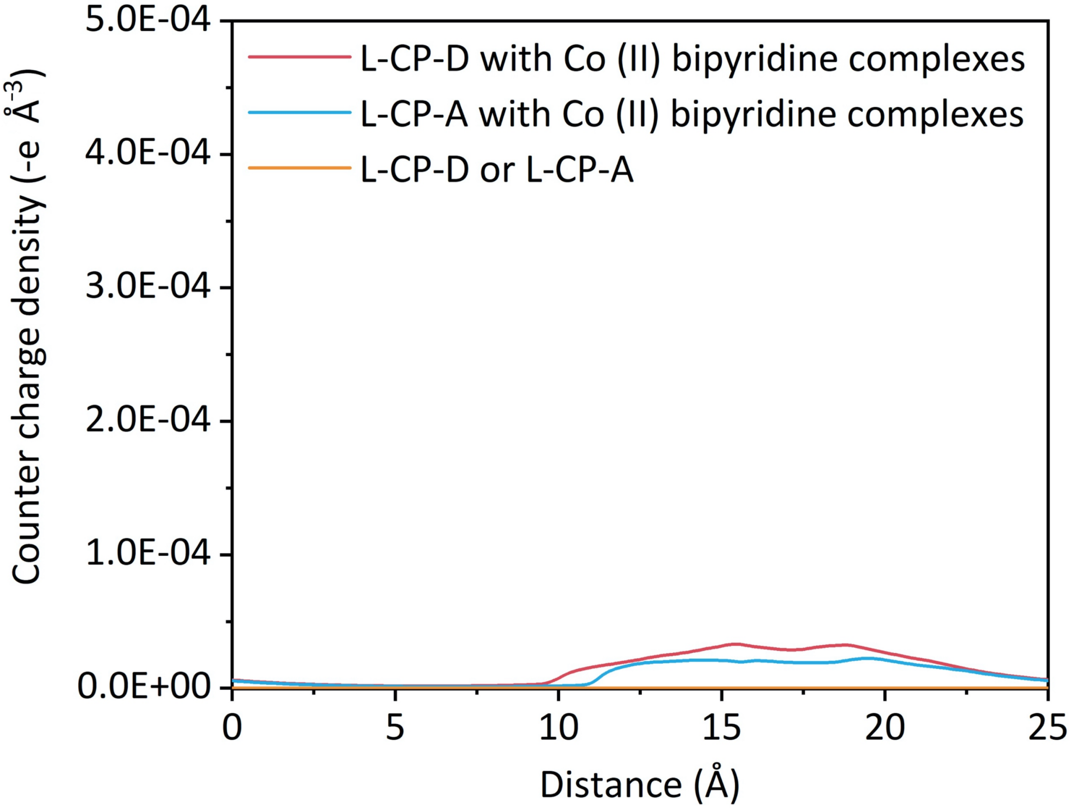

Supplementary Figure 38. Countercharge density distribution in Co (II) bipyridine complexes/L-CP-D and Co (II) bipyridine complexes /L-CP-A, before and after Co (II) bipyridine complexes adsorption.The linear PB distribution of the counter charge was turned to be a reasonable model.

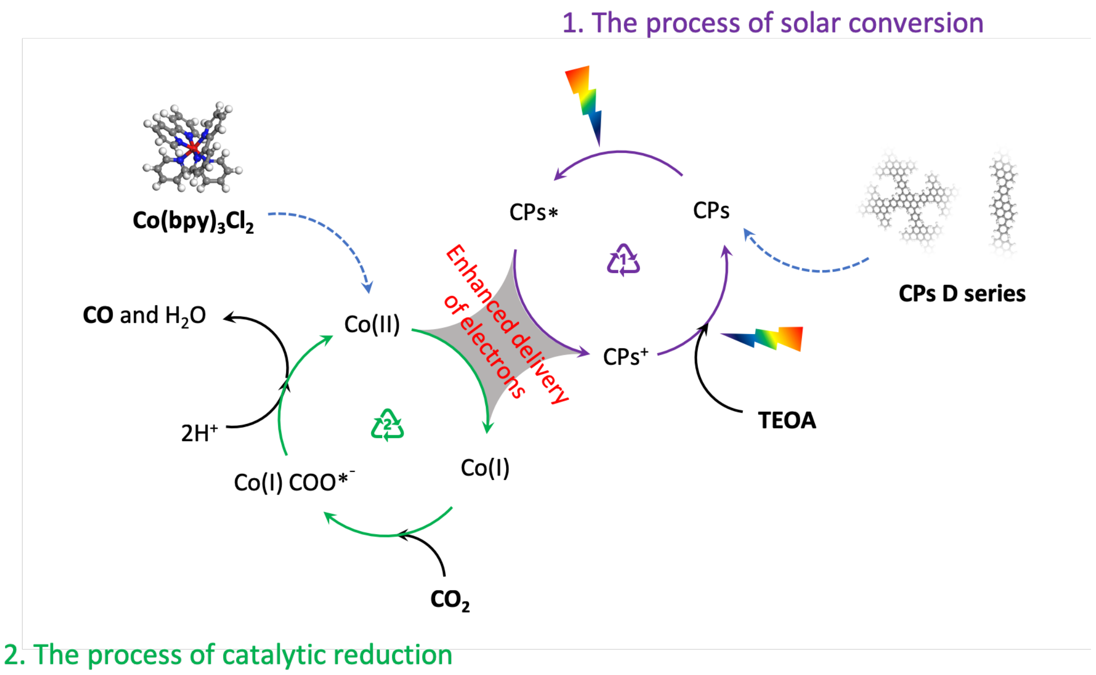

Supplementary Figure 39. Mechanism of two-step CO_2 photoreduction depended on enhanced delivery of electrons in CPs D series system.

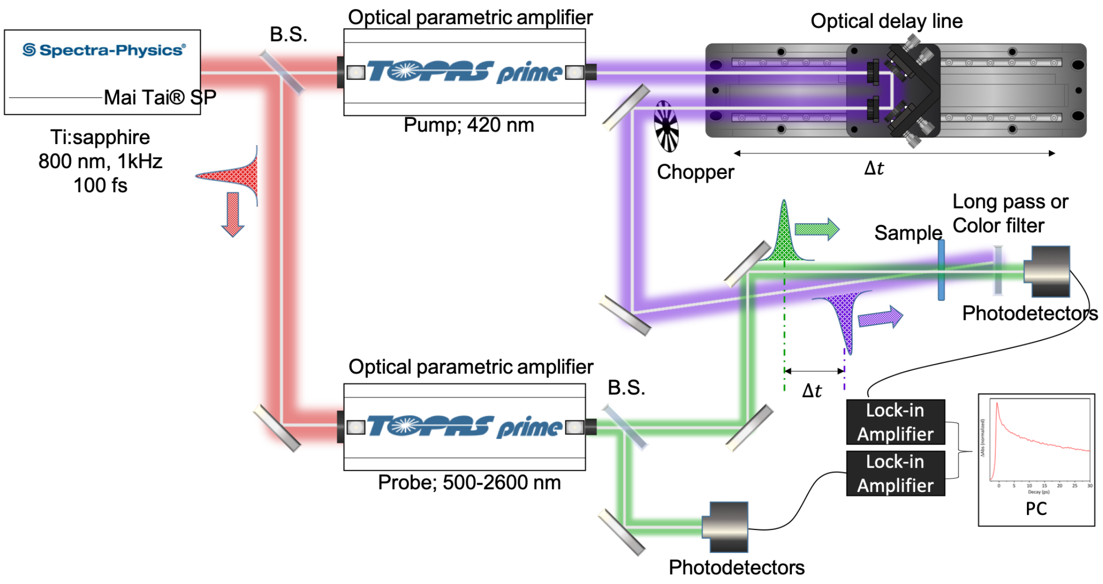

Supplementary Figure 40. The illustration of transient adsorption measurement.

Supplementary Tables

Supplementary Table 1. The energy levels within the L-CP-D, L-CP-A, N-CP-D and N-CP-A.

	L-CP-D	N-CP-D	L-CP-A	N-CP-A
Band gap	2.62 eV	2.47 eV	2.10 eV	1.98 eV
HOMO	1.41 V	1.36 V	1.47 V	1.43 V
LUMO	-1.21 V	-1.11 V	-0.63 V	-0.55 V

Supplementary Table 2. The energy levels within the L-CP-D, L-CP-A, N-CP-D and N-CP-A obtained from the UPS and Mott-Schottky tests.

		L-CP-D	N-CP-D	L-CP-A	N-CP-A
	Band gap	2.62 eV	2.47 eV	2.10 eV	1.98 eV
UPS test	HOMO	1.34 V	1.23 V	1.46 V	1.45 V
	LUMO	-1.28 V	-1.24 V	-0.64 V	-0.53 V
M-S test	HOMO	1.37 V	1.35 V	1.44V	1.43 V
	LUMO	-1.25 V	-1.12 V	-0.66 V	-0.55 V

Supplementary Table 3. The computed interlayer interaction energies (ΔE_i, J m^{-1}, and J m^{-2}) and distances ($D_{interlayer}$, Å) of interlayer for L-CP-D, L-CP-A, N-CP-D and N-CP-A.

Stacking		AA	AB	AB′	AC
ΔE_i (10^{-10}J m^{-1})	L-CP-D	-	-0.934	-0.925	-
	L-CP-A	-0.630	-0.713	-0.756	-
ΔE_i (J m^{-2})	N-CP-D	-	-0.116	-0.114	-
	N-CP-A	-0.058	-0.063	-0.063	-0.043
$D_{interlayer}$ (Å)	L-CP-D	-	3.53	3.53	-
	L-CP-A	3.82	3.59	3.51	-
	N-CP-D	-	3.35	3.60	-
	N-CP-A	3.84	3.73	3.74.	3.62

Supplementary Table 4. Comparison of CO_2 photoreduction performance of the reported catalysts in the system similar to this work.

Catalyst	Cocatalyst	Sacrificial agent	Major products evolution rate	Enhancement after modification*	Selectivity	AQY	Ref.
**L-CP-D**	**$Co(bpy)_3Cl_2$**	**TEOA**	**CO: 806 μmol $g^{-1}h^{-1}$**	**81-times**	**86.0 %**	3.39 %	**This work**
**N-CP-D**	**$Co(bpy)_3Cl_2$**	**TEOA**	**CO: 2274 μmol $g^{-1}h^{-1}$**	**138-times**	**82.0 %**	1.23 %	**This work**
$ZnIn_2S_4$-In_2O_3	$Co(bpy)_3Cl_2$	TEOA	CO: 3075 μmol $g^{-1}h^{-1}$	3.5-times	~79.4 %	/	1
In_2S_3-$CdIn_2S_4$	$Co(bpy)_3Cl_2$	TEOA	CO: 825 μmol $g^{-1}h^{-1}$	12.0-times	~73.3 %	/	2
CPs-BT	$Co(bpy)_3Cl_2$	TEOA	CO: 1213 μmol $g^{-1}h^{-1}$	4.6-times	81.6 %	1.75 %	3
CdS-BCN	$Co(bpy)_3Cl_2$	TEOA	CO: 250 μmol $g^{-1}h^{-1}$	10.3-times	81.1 %	/	4
BCN	$Co(bpy)_3Cl_2$	TEOA	CO: 94 μmol $g^{-1}h^{-1}$	/	76.2 %	/	5
HR-CN	$Co(bpy)_3Cl_2$	TEOA	CO: 297 μmol $g^{-1}h^{-1}$	22.3-times	96.7 %	/	6
MCN/CoO_x	$Co(bpy)_3Cl_2$	TEOA	CO: 204 μmol $g^{-1}h^{-1}$	2.76-times	78.5 %	0.25 %	7
CNU-BA	$Co(bpy)_3Cl_2$	TEOA	CO: 1036 μmol $g^{-1}h^{-1}$	15.0-times	81.8 %	/	8
MOF-525-Co	/	TEOA	CO: 201 μmol $g^{-1}h^{-1}$	3.13-times	/	/	9

*** The enhancement after modification were mentioned here due to the different researchers employed various evaluate system to measure the evolution rate.**

Supplementary Table 5. Residual palladium and copper metals detected by the inductively coupled plasma atomic emission spectroscopy (ICP-AES).

	L-CP-D	N-CP-D	L-CP-A	N-CP-A
Pd (wt.%)	0.453	0.661	0.553	0.824
Cu (wt.%)	N/A	N/A	N/A	0.005

Supplementary Table 6. The loading content of Co species detected by the inductively coupled plasma atomic emission spectroscopy (ICP-AES).

	L-CP-D	N-CP-D	L-CP-A	N-CP-A
Co (wt.%)	0.862	3.614	0.526	1.548

Supplementary Table 7. Apparent BET surface areas for the CPs

	L-CP-D	N-CP-D	L-CP-A	N-CP-A
BET surface area ($m^2 \cdot g^{-1}$)	85.35	827.84	15.81	466.07

Supplementary Table 8. Parameters of the time-resolved photoluminescence decay curves according to a biexponential decay in different atmosphere.

	In argon atmosphere					In CO_2 atmosphere				
	τ_1(ns)	A_1 (%)	τ_2 (ns)	A_2 (%)	τ_{av} (ns)	τ_1(ns)	A_1 (%)	τ_2 (ns)	A_2 (%)	τ_{av} (ns)
L-CP-D	0.242	45.8	2.606	54.2	1.523	0.126	78.6	0.840	21.4	0.279
N-CP-D	0.208	35.3	3.462	64.7	2.313	0.168	82.2	1.316	17.8	0.372
L-CP-A	0.105	83.8	0.792	16.2	0.216	0.123	85.2	0.941	14.8	0.244
N-CP-A	0.107	84.3	0.881	15.7	0.229	0.124	84.9	1.023	15.1	0.259

Supplementary methods

Computational methods.

All the computations were performed with the Vienna ab initio simulation package (VASP 5.4.4 version)[10,11], patched to include the implicit solvent VASPsol[12,13]. The electron exchange and correlation were handled with generalized gradient approximation using PBE functional and HSE06[14,15], and which were applied for structure optimization and electronic structures computations, respectively. The Grimme PBE-D3 and HSE06-D3 methods were used for the dispersion correction[16]. The plane wave cut-off energy was set to 400 eV, the electronic self-convergence was 10^{-6} eV, and the core electrons were replaced by the projector augmented wave pseudopotential[17,18]. The geometries are converged to a residual force smaller than 0.03 eV/Å using PBE-D3.

The L-CP-D, L-CP-A, N-CP-D, and N-CP-A were modeled in a monolayer structure, with a vacuum thickness of 25 Å. The atomic position and lattice parameters are fully relaxed during the slab mode optimized. The optimized lattice parameters were listed in Table S1, a = b = 11.44 Å, γ = 100.65 degree for N-CP-D, a = b = 16.03 Å, γ = 79.77 degree for N-CP-A, respectively. And the value was a = 16.14 Å, b = 21.27 Å, γ = 90.22 degree and a = 11.96 Å, b = 22.10 Å, γ = 89.60 degree for L-CP-A and L-CP-D, respectively. The optimization structures were shown in Figure S1. The Brillouin zone was integrated by 3×1×1, 3×1×1, 3×3×1, and 3×3×1 Monkhorst-Pack grid for optimization for L-CP-D, L-CP-A, N-CP-D, and N-CP-A, respectively. There existed a dihedral angle around 50 degrees between benzene and pyrene rings, due to the steric effect for L-CP-D and N-CP-D, as shown in Figure S1a and S1c, which was reported in similar structures[19]. And the optimized L-CP-A and N-CP-A were flat structures. In addition, the band structures were calculated along the high symmetry lines G (0.0, 0.0, 0.0)-F (0.0, 0.5, 0.0)-Z (0.0, 0.0, 0.5)-B (0.5, 0.0, 0.0) using HSE06-D3. The calculated bandgaps were 2.62 eV, 1.84 eV, 2.45 eV and 1.29 eV for L-CP-D, L-CP-A, N-CP-D, and N-CP-A, respectively. The adsorption energies of the Co (II) bipyridine complexes on L-CP-D or L-CP-A surface were calculated via $\Delta E_{bind} = E(Co(bpy)_2^{2+}/surf) - E(Co(bpy)_2^{2+}) - E(surf)$, where $E(Co(bpy)_2^{2+}/surf)$ denoted the energies of $Co(bpy)_2^{2+}$/L-CP-D or $Co(bpy)_2^{2+}$/L-CP-A, $E(Co(bpy)_2^{2+})$ represented the energies of $Co(bpy)_2^{2+}$ and E(surf) stood for the energy of L-CP-D and L-CP-A surface. The

interlayer interaction energies of L-CP-D, L-CP-A, N-CP-D and N-CP-A were calculated via, ΔEi =(E(bilayer) -2×E(monolayer))/A, where E was the computational total energy of L-CP-D, L-CP-A, N-CP-D and N-CP-A, and A was the surface area of N-CP-A and N-CP-A, and A was the length of L-CP-A and L-CP-D, which was the lattice parameter a.

Photocatalytic cyclic testing experiment and AQY Evaluation.

For cyclic testing experiment, the 25 mL of fresh solution (acetonitrile/water = 7:3) and 5 mL of triethanolamine were added into the Pyrex glass reaction cell. After complete evacuation of the reaction system, ~80 kPa of pure CO_2 gas was injected into the airtight system. The subsequent photoirradiation and sampling analysis processes are consistent with the previous photocatalytic measurement process. The apparent quantum efficiency (AQY) was measured under similar conditions as described above except for the light intensity and the wavelength regions of the irradiation light. A series of band-pass filters were adopted to control the wavelength regions of incident light. A water filer was also used together with the band-pass filter to avoid the possible damage caused by heating effect of Xe lamp. The average intensity of irradiation was determined by using a spectroradiometer (AvaSolar-1, Avantes, America). The AQY at each monochromatic wavelength was calculated by following the equation below:

$$\text{AQY for CO evolution} = N(CO) \times 2 / N(\text{photons}) \times 100\%$$

EPR measurement.

N-CP-D powder with Co complexes was obtained by an adsorption process and then place it into the EPR sample tube. A degassing device was used to make the tube oxygen-free and seal the sample tube by sintering the nozzle. After these pre-treatments, the sample was tested before and after irradiation with an electron spin resonance (JES-FA200, JEOL, Japan) spectrometer.

TA measurement.

As shown in Figure S40, both excitation light and probe lights were focused and overlapped on the surface of the photocatalysts. The transmitted probe light was focused into the Si or InGaAs photodetectors with a detection range of 500-1000 and 1000-2600 nm. Scattered excitation light was blocked by a long wave pass filter placed in front of the photodetectors. The intensity of the transmitted probe light T is detected by the amplification of the signal from the probe light by a lock in amplifier synchronized with the 1kHz repetition of the probe light pulsed. The change of the

intensity of the transmitted probe light caused by the excitation light $\Delta T(t)$ was detected by the differential amplification of the signal from probe light and that from the reference light using a lock-in amplifier synchronized with the frequency of the optical chopper, where t is the time delay between pump and probe pulses generated by optical delay line. For the measurement of transient absorption spectra, the wavelength of the probe light was measured over the range from 500 to 2600 nm, while the measurement for the signal decay of the transient absorption was performed by scanning the delay time to about 1 ns as maximum using optical delay line.

Supplementary Discussion

The CO_2 photoreduction under different conditions

As shown in Figure 22, except for using the same amount of isolated cobalt chloride or dipyridyl instead of Co (II) bipyridine complexes as the cocatalyst, we also performed CO_2 photoreduction over N-CP-D or L-CP-D. The lower performance revealed that only assembled Co (II) bipyridine complexes act the efficient site for the CO_2 photoreduction. As shown in Figure 23, the selectivity of the amount of cocatalyst to the catalytic products formed over the CPs-D series was performed. Both L-CP-D and N-CP-D presented weak activities without a cocatalyst, whereas their H_2 evolution rate under this condition was relatively high. As detected by the inductively coupled plasma mass spectrometry (ICP-MS), the formation of residual palladium and copper metals, which may act as active sites for H_2 evolution, during the synthesis process might account for this consequence (Table S5). The production of H_2 decreased with an increasing amount of cocatalyst, while the production of CO gradually increased. The increase in CO production over L-CP-D was not apparent after adding more than 1 μmol of cocatalyst, and this phenomenon appeared in the N-CP-D system after adding more than 5 μmol of cocatalyst. With the addition of 10 μmol of cocatalyst, the CO evolution selectivity of L-CP-D and N-CP-D obtained through our experiment reached 86% and 82%, respectively. In addition, the ICP-AES measurement was employed to determine the loading amount of the Co species on the CPs (Table S6) when adding with 5 μmol of Co (II) bipyridine complexes cocatalyst. The main reason of improved activities over CPs-D series is attribute to the enhanced intermolecular conjugate interactions.

The thermal stability of CPs

As shown in Figure S25, the relatively high pyrolysis temperature determined from thermogravimetric analysis (TGA) also provides evidence for the stability.

The spin density of CPs

As shown in Figure 37, a spin-polarized and implicit solvation computation was used to model the case of $Co(bpy)_3^{2+}$, $Co(bpy)_2^{2+}$, $Co(bpy)_2^{2+}$/L-CP-D and $Co(bpy)_2^{2+}$/L-CP-A. The GGA+U approximation with the Dudarev "+U" term with a U-J value of 3.0 eV for the d electrons of Co atom, since GGA cannot correctly describe the electronic structure of Co(II)[20]. Implicit solvent through Poisson-Boltzmann (PB) continuum

approximation[21,22], was applied to consider solvent effect, which was implemented by Hennig group in VASPsol[3,4].The cavity surface tension was set to zero to obtain numerical stability, and the Debye length was set to 3.0 Å to consider the electric-field screening effect in electrolyte solvent[23]. Here the L-CP-D and L-CP-A were built by a (2×1×1) supercell, and single gamma-point grid sampling was employed for Brillouin zone integration. The electron configuration of Co^{2+} was calculated to be triplet states, similar to the reported[24,25]. And the spin density distributions in $Co(bpy)_2^{2+}$/L-CP-D and $Co(bpy)_2^{2+}$/L-CP-A, was presented in Figure S3, which was triplet states. The $Co(bpy)_3^{2+}$ was tended to lose a bpy molecular, providing more vacancy for the reaction. The $Co(bpy)_2^{2+}$ was clarified to be stronger adsorbed on L-CP-D than on L-CP-A, with the adsorption energy of -0.70 eV and -0.60 eV respectively.

Supplementary References

1. Wang, S., Guan, B. Y. & Lou, X. W. D. Construction of $ZnIn_2S_4$-In_2O_3 hierarchical tubular heterostructures for efficient CO_2 photoreduction. *J. Am. Chem. Soc.* **140**, 5037-5040 (2018).

2. Wang, S., Guan, B. Y., Lu, Y. & Lou, X. W. D. Formation of hierarchical In_2S_3-$CdIn_2S_4$ heterostructured nanotubes for efficient and stable visible light CO_2 reduction. *J. Am. Chem. Soc.* **139**, 17305-17308 (2017).

3. Yang, C., Huang, W., da Silva, L. C., Zhang, K. A. & Wang, X. Functional conjugated polymers for CO_2 reduction using visible light. *Chem-A Eur. J.* **24**, 17454-17458 (2018).

4. Zhou, M., Wang, S., Yang, P., Huang, C. & Wang, X. Boron carbon nitride semiconductors decorated with CdS nanoparticles for photocatalytic reduction of CO_2. *ACS Catal.* **8**, 4928-4936 (2018).

5. Huang, C. *et al.* Carbon-doped BN nanosheets for metal-free photoredox catalysis. *Nat. Commun.* **6**, 7698 (2015).

6. Zheng, Y., Lin, L., Ye, X., Guo, F. & Wang, X. Helical graphitic carbon nitrides with photocatalytic and optical activities. *Angew. Chem. Int. Edit.* **53**, 11926-11930 (2014).

7. Lin, J., Pan, Z. & Wang, X. Photochemical reduction of CO_2 by graphitic carbon nitride polymers. *ACS Sustain. Chem. Eng.* **2**, 353-358 (2013).

8. Qin, J., Wang, S., Ren, H., Hou, Y. & Wang, X. Photocatalytic reduction of CO_2 by graphitic carbon nitride polymers derived from urea and barbituric acid. *Appl. Catal. B- Environ.* **179**, 1-8 (2015).

9. Zhang, H. *et al.* Efficient Visible-Light-Driven Carbon Dioxide Reduction by a Single-Atom Implanted Metal–Organic Framework. *Angew. Chem. Int. Ed.***55**, 14310-14314 (2016).

10. Kresse, G. & Hafner, J. Ab initio molecular dynamics for liquid metals. *Phys. Rev. B* **47**, 558-561 (1993).

11. Kresse, G. & Furthmüller, J. Efficient iterative schemes for ab initio total-energy calculations using a plane-wave basis set. *Phys. Rev. B* **54**, 11169-11186 (1996).

12. Mathew, K. *et al.* Implicit solvation model for density-functional study of nanocrystal surfaces and reaction pathways. *J. Chem. Phys.* **140**, 084106 (2014).

13. Mathew, K. & Richard G. H. Implicit self-consistent description of electrolyte in plane-wave density-functional theory. *arXiv preprint arXiv.* **1601**, 03346 (2016).

14. Perdew, J. P. Kieron B, & Matthias E. Generalized gradient approximation made simple. *Phys. Rev. Lett.* **77**, 3865 (1996).

15. Heyd, J., Scuseria, G. E. & Ernzerhof, M. Hybrid functionals based on a screened Coulomb potential. *J. Chem. Phys.* **118**, 8207-8215 (2003).

16. Grimme, S. Semiempirical GGA-type density functional constructed with a long-range dispersion correction. *J. Comput. Chem.* **27**, 1787-1799 (2006).

17. Kresse, G. & Joubert, D. From ultrasoft pseudopotentials to the projector augmented-wave method. *Phys. Rev. B* **59**, 1758-1775 (1999).

18. Blöchl, P. E. Projector augmented-wave method. *Phys. Rev. B* **50**, 17953-17979 (1994).

19. Wang, L. *et al.* Conjugated Microporous Polymer Nanosheets for Overall Water Splitting Using Visible Light. *Advanced Materials* **29**, 1702428 (2017).

20. Yang, S. et al. Oxygen-vacancy abundant ultrafine Co3O4/graphene composites for high-rate supercapacitor electrodes. *Adv. Sci.* **5**, 1700659 (2018).

21. Gunceler, D. *et al.* The importance of nonlinear fluid response in joint density-functional theory studies of battery systems. *Model. Simul. Mater. Sc.* **21**, 074005 (2013).

22. Letchworth-Weaver, K. & Arias, T. A. Joint density functional theory of the electrode-electrolyte interface: Application to fixed electrode potentials, interfacial capacitances, and potentials of zero charge. *Phys. Rev. B* **86**, 075140 (2012).

23. Wang, P., Steinmann, S. N., Fu, G., Michel, C. & Sautet, P. Key Role of Anionic Doping for H_2 Production from Formic Acid on Pd (111). *ACS Catal.* **7**, 1955-1959, (2017).

24. Sreekantan Nair Lalithambika, S., Golnak, R., Winter, B. & Atak, K. Electronic Structure of Aqueous $[Co(bpy)_3]^{2+/3+}$ Electron Mediators. *Inorg. Chem.* **58**, 4731-4740, (2019).

25. Vargas, A., Zerara, M., Krausz, E., Hauser, A. & Lawson Daku, L. M. Density-functional theory investigation of the geometric, energetic, and optical properties of the cobalt(II)tris(2,2'-bipyridine) complex in the high-spin and the jahn-teller active low-spin states. *J. Chem. Theo. Comput.* **2**, 1342-1359, (2006).
